# Distal enhancer-insulator module of *GDF6* is essential for cochlear formation

**DOI:** 10.1172/jci.insight.195462

**Published:** 2026-06-18

**Authors:** Mohammad Faraz Zafeer, Clemer Abad, Havva Ortabozkoyun, Memoona Ramzan, Guney Bademci, Maria C. Robayo, Duygu Duman, Rolen M. Quadros, Shengru Guo, Juan I. Young, Anthony J. Griswold, Channabasavaiah B. Gurumurthy, Derek M. Dykxhoorn, Katherina Walz, Mustafa Tekin

**Affiliations:** 1 John P. Hussman Institute for Human Genomics,; 2 Dr. John T. Macdonald Foundation Department of Human Genetics, and; 3 Sylvester Comprehensive Cancer Center, University of Miami Miller School of Medicine, Miami, Florida, USA.; 4 Howard Hughes Medical Institute, Miller School of Medicine, University of Miami, Miami, Florida, USA.; 5 Department of Audiology, Ankara University Faculty of Health Sciences, Ankara, Turkey.; 6 Ankara University Rare Diseases Application and Research Center, Ankara, Turkey.; 7 Mouse Genome Engineering Core Facility, University of Nebraska Medical Center, Omaha, Nebraska, USA.; 8 Department of Cell and Molecular Biology, University of Mississippi Medical Center, Jackson, Mississippi, USA.; 9 Departamento de Ingeniería Biomédica, Universidad Austral, CONICET, Pilar, Argentina.

**Keywords:** Development, Genetics, Genetic diseases, Molecular genetics, Stem Cells

## Abstract

Several genes guide inner ear development, and mutations in these genes can cause malformations that result in congenital hearing loss. However, the contribution of noncoding regulatory elements remains largely unclear. This study investigates the function of distal enhancer elements in the transcriptional regulation of *GDF6*, a gene implicated in cochlear development. Using mouse models with targeted deletions, human inner ear organoids, and CRISPR interference (CRISPRi), we identified a downstream regulatory interval harboring a developmental enhancer required to maintain *GDF6* expression during otic epithelial maturation and cochlear morphogenesis. Deletion of this regulatory region or targeting of CRISPRi-based repressors to these regions resulted in decreased *GDF6* expression, failure of otic-epithelium development, and prevention of hair cell–like differentiation, reflecting cochlear aplasia observed in patients with corresponding genomic deletions. These findings highlight the contribution of long-range regulatory elements to auditory development and illustrate how their disruption contributes to human deafness.

## Abstract 2

A distant DNA switch controlling the *GDF6* gene is required for inner ear formation; its loss causes complete absence of the cochlea and deafness.

## Graphical abstract

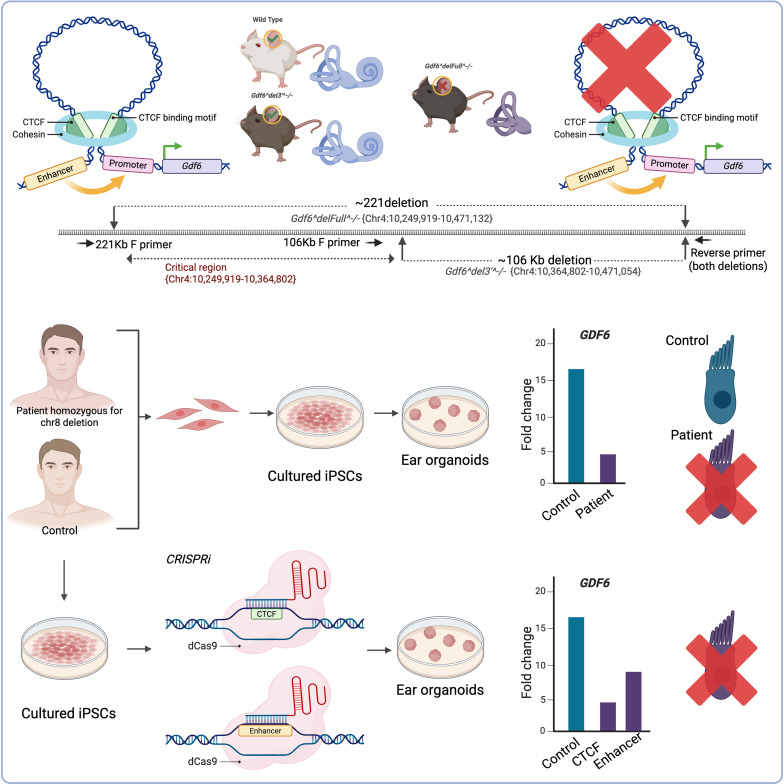

## Introduction

The mammalian inner ear is a complex structure that develops through a cascade of morphogenetic events originating from the embryonic otic vesicle and resulting in a dorsal vestibular system and a ventral auditory apparatus, the cochlea (1). Mechanosensory hair cells and supporting cells are intricately organized within the cochlea to transduce sound into neural signals (1–3). Proper cochlear development requires tightly regulated spatiotemporal gene expression to pattern the otic epithelium and surrounding tissues (4). Disruption of these early developmental processes can lead to congenital hearing loss, one of the most common sensory deficits in humans, affecting approximately 1 in 500 newborns (5).

Mutations in approximately 30 genes have been linked to cochlear malformations in humans, underscoring our limited understanding of cochlear developmental genetics (5). Notable examples include mutations in *FGF3* that lead to complete inner ear agenesis (6) and mutations in *FOXF2* and *GREB1L* that result in cochlear hypoplasia and deafness (5, 7). However, many cases of cochlear malformations lack mutations in known coding genes, suggesting that noncoding regulatory region defects may be an underrecognized contributor.

During embryogenesis, precise gene regulation is often achieved by long-range cis-regulatory elements, or enhancers, that activate transcription in specific cell types and developmental windows. Enhancers can reside hundreds of kilobases away from their target genes, yet communicate with promoters via chromatin looping and higher-order genome architecture (8). This regulatory paradigm allows “professed developmental regulator genes,” such as transcription factors and morphogens, to integrate multiple spatial cues (9). A prominent example of such complex regulation is X-linked deafness in *DFN3,* caused by multi-kilobase deletions upstream of *POU3F4* that remove critical enhancers required for inner ear development (10). In these cases, the pathogenic variant lies in a noncoding region of DNA that controls the expression of key developmental genes. Such disorders have been termed “enhanceropathies” (11), highlighting a mechanism through which enhancer dysregulation can lead to congenital disease (12). One of the difficulties in classifying the pathogenicity of these variants is the relatively poor annotation of noncoding regions of the genome compared with coding variants. As such, comprehensive functional studies are needed to link noncoding changes to developmental phenotypes (8).

Growth differentiation factor 6 (*GDF6*), also known as *BMP13*, is a key developmental gene that encodes a TGF-β superfamily ligand, regulating multiple aspects of embryogenesis (13). In mice, *Gdf6* is expressed in the developing inner ear, joints, and retina (1, 14–18). In humans, rare monoallelic coding variants in *GDF6* have been reported in patients with joint fusions (Klippel-Feil syndrome) and ocular anomalies (9, 15). We have recently reported that approximately 200 kb homozygous deletions (Chr8:96,596,661–96,803,788; hg19), located in a gene desert approximately 350 kb downstream of *GDF6,* are associated with nonsyndromic deafness and cochlear aplasia (1). The *GDF6* coding region remained intact in these individuals, suggesting that the distant noncoding deletions are likely the cause of deafness. Patient-induced pluripotent stem cell–derived (iPSC-derived) otic cultures exhibited substantially reduced *GDF6* expression, and a *Gdf6*-KO mouse model showed cochlear aplasia that closely mirrored the human phenotype (1). These findings support a role for *GDF6* among the genes that contribute to early cochlear development.

In this study, we generated a mouse model carrying an approximately 200 kb deletion that mirrors the human deletion associated with cochlear aplasia. A smaller, partial deletion did not reproduce the phenotype, thereby narrowing the critical region to approximately 100 kb. We then differentiated human iPSCs into otic progenitors under 2D conditions. We used single-cell assay for transposase-accessible chromatin with sequencing (scATAC-Seq) to assess chromatin accessibility at the cis-regulatory region of the *GDF6* and identified that the region indeed has active areas of accessibility. In further studies, we used inner ear organoids (IEOs) from a control (CD2) and a patient (166-101) with (Chr8:96,596,661–96,803,788; hg19), an approximately 200 kb downstream deletion. Patient IEOs showed reduced *GDF6* expression and impaired hair cell–like cell differentiation. Bulk ATAC-Seq and ChIP-Seq analyses of IEOs identified regulatory regions with CTCF binding and dynamic enhancer activation during otic-epibranchial progenitor domain formation. CRISPRi targeting the region containing CTCF and the enhancer site in control iPSCs reduced *GDF6* expression and disrupted the developmental trajectory of IEOs, imitating patient and mouse enhancer deletion phenotypes. Together, these findings define a regulatory architecture in which *GDF6* expression depends on regulatory elements within a sub-topologically associating domain that acts as a developmental maintenance (or stabilization) enhancer, and disruption of this topology by genetic or epigenetic factors leads to cochlear malformation and hearing loss.

## Results

### Deletion of Gdf6 regulatory region results in deafness, cochlear aplasia, and reduced Gdf6 expression in mouse models.

**Figure 1:**
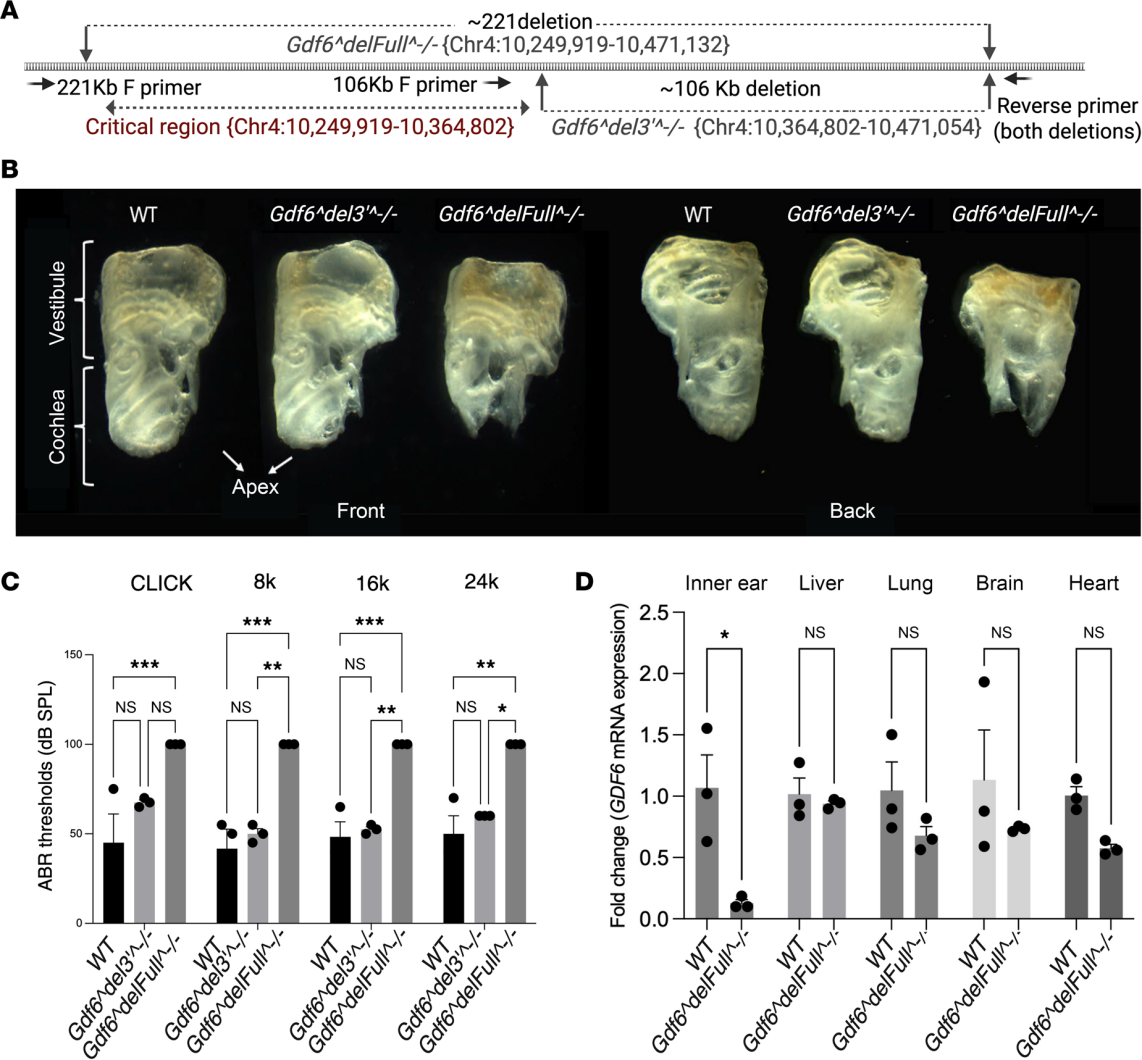
Characterization of *Gdf6^delFull–/–^* and *Gdf6^del3′–/–^* mice. (**A**) Schematic of CRISPR/Cas9 sgRNA placement and the deleted regions in *Gdf6^delFull–/–^* (~221 kb; Chr4:10,249,919–10,471,132) and *Gdf6*^del3*′*–/–^ (~106 kb; Chr4:10,364,802–10,471,054) mice, with the critical region (Chr4:10,249,919–10,364,802) indicated. Positions of the 221 kb forward, 106 kb forward, and common reverse genotyping primers are shown. (**B**) Whole-mount front and back views of the inner ear from WT, *Gdf6*^del3*′*–/–^, and *Gdf6^delFull–/–^* mice, showing preserved vestibular and cochlear structures in WT and *Gdf6*^del3*′*–/–^, and cochlear aplasia in *Gdf6^delFull–/–^* mice (*n* = 3 per genotype). The cochlear apex is indicated by arrows. (**C**) Auditory brainstem response (ABR) thresholds for click stimuli and tone bursts at 8, 16, and 24 kHz. *Gdf6^delFull–/–^* mice showed markedly elevated thresholds compared with WT mice across all frequencies (***P < 0.001 at CLICK, 8 kHz, and 16 kHz; **P < 0.01 at 24 kHz) and compared with *Gdf6*^del3*′*–/–^ mice (**P < 0.01 at 8 kHz and 16 kHz; *P < 0.05 at 24 kHz; ns at CLICK), consistent with profound hearing impairment. Thresholds did not differ significantly between WT and *Gdf6*^del3*′*–/–^ mice at any frequency (ns; *n* = 3 per genotype). (**D**) qRT-PCR analysis of Gdf6 mRNA (normalized to *Gapdh*) at E17 in inner ear, liver, lung, brain, and heart from *Gdf6^delFull–/–^* and WT embryos (*n* = 3 per genotype). A significant reduction in Gdf6 expression is restricted to the inner ear (*P < 0.05); all other tissue comparisons are nonsignificant (ns). Data are presented as mean ± SEM. Statistical comparisons were performed by 2-way ANOVA followed by Tukey’s post hoc test for multiple comparisons; only comparisons reaching statistical significance are annotated; all others are labeled ns.

To investigate the functional role of *Gdf6* enhancers in cochlear development, 2 mouse models were successfully generated using CRISPR/Cas9, each with deletions of approximately 106 kb and approximately 221 kb downstream of *Gdf6* (Figure 1A and Supplemental Figures 2–5; supplemental material available online with this article; https://doi.org/10.1172/jci.insight.195462DS1). Auditory brainstem response (19) testing at 5 months of age revealed normal hearing thresholds in *Gdf6^del3′–/–^* mice, indicating that the approximately 106 kb deletion did not impair auditory function. In contrast, *Gdf6^delFull–/–^* mice exhibited profound deafness across all tested frequencies, including click stimuli and pure-tone bursts at 8, 16, and 24 kHz (Figure 1C). Whole-mount imaging of the inner ear at P5 demonstrated that although *Gdf6^del3′–/–^* mice displayed cochlear structures comparable to WT mice, *Gdf6^delFull–/–^* mice presented with cochlear aplasia, characterized by the complete absence of the cochlear duct (Figure 1B). The vestibular region appeared morphologically intact in both deletion models, indicating that the deleted enhancer regions are required for cochlear development, and vestibular structures appeared morphologically intact. The same phenotype was observed in *Gdf6-*KO mice (1). Concurring with this preliminary result, analysis of *Gdf6* mRNA expression at E17 revealed a significant reduction in the inner ear of *Gdf6^delFull–/–^* mice compared with WT controls (Figure 1D). In contrast, *Gdf6* expression levels in other tissues, including the brain, heart, lung, and liver, were comparable between *Gdf6^delFull–/–^* and WT mice (*n* = 3 biological replicates per genotype), and the nearby genes were unaffected (Supplemental Figure 1). These findings identified a genomic region containing a regulatory enhancer required for cochlear development that maintains appropriate spatiotemporal levels of Gdf6 expression in the inner ear.

Previously, deletions were observed in 2 families with cochlear aplasia that coincided with a long noncoding RNA (*CFAP418-AS1*) located on chromosome 8. The mouse ortholog of this RNA is *1700123O12Rik* (Supplemental Figure 2), which is situated outside the critical region (Chr4:10,249,919–10,364,802), excluding it as a probable cause of the observed phenotype.

### scATAC-Seq reveals the presence of GDF6-associated open chromatin elements in the deletion region (Chr8:96,596,661–96,803,788;hg19).

To characterize cellular composition and gene-expression changes in patient (166-101) versus control (CA-26) samples, we first performed single-cell RNA-Seq (scRNA-Seq) on day 11 2D otic progenitor cells derived from human iPSCs. This analysis identified GDF6-expressing cell populations in the control sample that were reduced in the patient sample (Supplemental Figure 7A).

Because the deleted interval is noncoding, and mouse studies indicated that the downstream cochlear regulatory region is required for Gdf6 expression during inner ear development, we next sought to identify candidate regulatory elements in the patient-derived otic progenitor model. To define regulatory elements controlling GDF6 expression during early otic progenitor differentiation, we performed scATAC-Seq on day 11 cultures. Comparative peak calling identified multiple open chromatin regions downstream of the GDF6 locus (chr8q22.1) in control cells (CA-26) that were absent in patient-derived cells due to the Chr8:96,596,661–96,803,788; hg19 deletion (Supplemental Figure 7). Among the most significantly altered peaks were 4 loci: chr8:95,648,167–95,648,667 (log_2_fold change [FC] = –5.29, FC = –39.2), chr8:95,681,386–95,681,886 (log_2_FC = –5.38, FC = –41.7), chr8:95,732,244–95,732,744 (log_2_FC = –3.70, FC = –13.0), and chr8:95,758,585–95,759,085 (log_2_FC = –5.93, FC = –60.9) (Supplemental Figure 7, B and C). These analyses were initially performed in 2D otic-lineage cultures rather than inner ear organoids to enable high-resolution mapping of chromatin accessibility differences between patient and control samples. The affected regions lie within an approximately 120 kb intergenic interval downstream of *GDF6*, consistent with the predicted locations of enhancer and insulator elements. Notably, the peak at chr8:95,758,585–95,759,085 overlaps a previously reported CTCF-binding site supported by Encyclopedia of DNA Elements (ENCODE) datasets available through the UCSC Genome Browser (20, 21), suggesting possible disruption of local chromatin insulation (Supplemental Figure 8). Loss of these accessible sites in the patient sample supports a model in which deletion of downstream regulatory elements may contribute to *GDF6* dysregulation. These 2D single-cell datasets were used primarily to map lineage-specific regulatory consequences of the deletion, and the candidate regulatory elements identified here were subsequently evaluated in the 3D inner ear organoid model.

### Loss of the GDF6 regulatory region/enhancer impairs hair cell differentiation.

**Figure 2:**
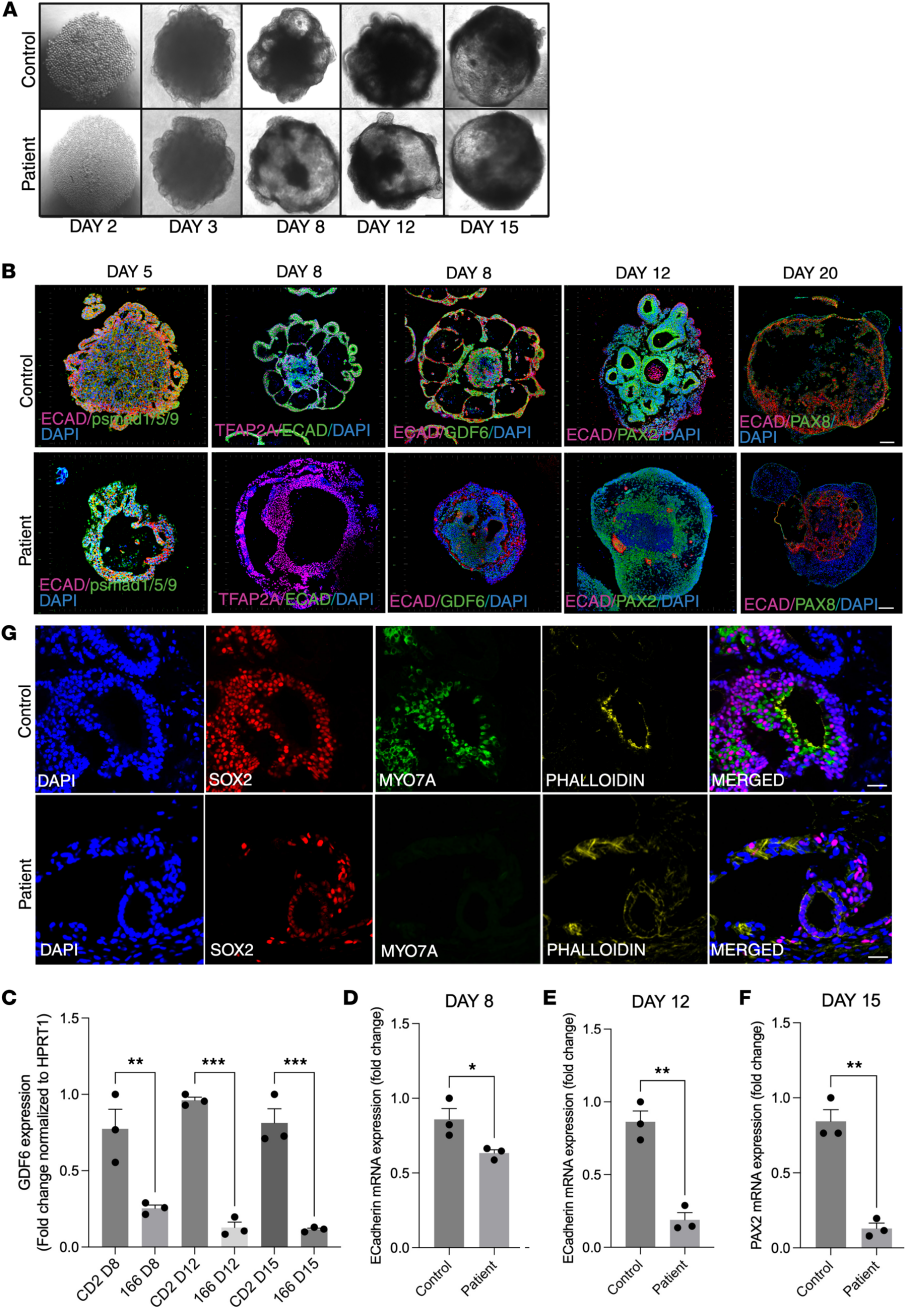
A putative *GDF6* enhancer lesion impairs otic organoid development and hair cell formation in patient-derived IEOs. (**A**) Brightfield images of inner ear organoids (IEOs) differentiated from control (CD-2) and proband (166-101) iPSCs at days –2, 3, 8, 12, and 15. (**B**) Representative confocal immunofluorescence images (*n* = 3, 20× magnification; scale: 50 µm) of control and proband IEOs across differentiation: day 5-ECAD, pSMAD1/5/9, Hoechst (blue); day 8-TFAP2A/AP2, CDH1/ECAD, DAPI (blue); day 8-CDH1/ECAD, GDF6, DAPI (blue); day 12-CDH1/ECAD, PAX2, DAPI (blue); day 20-CDH1/ECAD, PAX8, DAPI (blue). Proband IEOs show reduced and disorganized expression of otic epithelial and prosensory markers compared with controls. (**C**) qRT-PCR analysis of *GDF6* mRNA in control (CD2) and proband (166-101) IEOs at days 8, 12, and 15, normalized to *HPRT1*. Proband cultures show significantly reduced GDF6 expression at all 3 time points (**P < 0.01 at day 8; ***P < 0.001 at days 12 and 15). Statistical comparisons were performed by 1-way ANOVA followed by Tukey’s post hoc test for multiple comparisons (*n* = 3). (**D**) qRT-PCR analysis of *CDH1* (E-cadherin) mRNA expression at day 8, normalized to *HPRT1*, showing modestly reduced expression in proband IEOs (*P < 0.05; *n* = 3). (**E**) qRT-PCR analysis of *CDH1* mRNA expression at day 12, normalized to *HPRT1*, showing markedly reduced expression in proband IEOs (**P < 0.01; *n* = 3). (**F**) qRT-PCR analysis of PAX2 mRNA expression at day 15, normalized to *HPRT1*, showing significantly reduced prosensory marker expression in proband IEOs (**P < 0.01; *n* = 3). Statistical comparisons in panels **D**–**F** were performed by unpaired 2-tailed Student’s *t* test. (**G**) Representative confocal immunocytochemistry (*n* = 3; 40× magnification; scale bar: 20 µm) of day 55 IEO sections stained for SOX2 (red, supporting cells), MYO7A (green, hair cells), phalloidin (yellow, F-actin), and DAPI (blue, nuclei). Control IEOs contain robust MYO7A-positive hair cells with organized apical F-actin and SOX2-positive supporting cells, whereas proband IEOs lack MYO7A-positive cells and display reduced, disorganized SOX2 immunoreactivity.

Brightfield imaging across differentiation showed progressive morphological divergence between control and patient IEOs, with patient organoids displaying fewer and less-organized vesicle-like structures beginning at day 8 and persisting through day 15 (Figure 2A and Supplemental Figure 9). By day 5, both control and patient IEOs showed phosphorylated SMAD1/5/9 (pSMAD1/5/9) and E-cadherin–positive (CDH1/ECAD-positive) surface ectoderm/placodal epithelial features (Figure 2B and Supplemental Figure 10). We next assessed *GDF6* expression during early otic differentiation and found that it was significantly reduced in patient-derived cultures compared with controls at days 8, 12, and 15 (Figure 2C and Supplemental Figure 16). Consistent with these findings, TFAP2A (AP-2α) and ECAD/CDH1 expression was reduced in patient IEOs at day 8 (Figure 2D) and day 12 (Figure 2E), and paired box 2 (PAX2) expression was reduced by day 15 (Figure 2F), and PAX8 expression was diminished by day 20. Importantly, to assess the impact of enhancer deletion on hair cell–like cell differentiation, IEOs were cultured until day 55 and then subjected to immunocytochemical analysis for hair cell–like cells (Myosin VIIa, MYO7A), supporting cells (SOX2), and the actin cytoskeleton (phalloidin or Espin, ESPN). In the control, IEO, SOX2-positive supporting cells, and MYO7A-positive hair cells were well-organized around the lumen, with ACTIN localized at the apical surface of the hair cells (Figure 2G). In contrast, patient-derived IEO showed a striking reduction in MYO7A-positive cells, suggesting severe impairment in hair cell–like cell differentiation. Although SOX2-positive supporting cells were present, their distribution was less well-defined, with irregular patterns. This altered distribution may reflect defects in cell fate specification, migration, or structural organization, which could contribute to the observed impairment in hair cell–like cell differentiation and maturation in patient-derived IEOs. Phalloidin staining further confirmed structural deficiencies in the patient IEOs, indicating defective cytoskeletal organization in developing hair cell–like cell bundles (Figure 2G and Supplemental Figures 12 and 13). To support these observations, quantitative reverse-transcription PCR (qRT-PCR) analysis of sensory-lineage markers (*POU4F3, SOX2*) was performed and is shown in Supplemental Figure 11. Patient-derived IEOs exhibited levels consistent with impaired prosensory differentiation. Additional single-channel immunostaining for MYO7A, SOX2, and ESPN (Supplemental Figures 12 and 13) further demonstrated the disrupted organization of hair cell–like and supporting cells, confirming the differentiation deficits observed in Figure 2G.

The absence of MYO7A-positive hair cell–like cells showed a failure in hair cell differentiation, and the reduced SOX2-positive cells suggest impaired supporting cell development, phenotypes related to the cochlear aplasia observed in *Gdf6^delFull–/–^* mice (Figure 1B). It is important to note that under sonic hedgehog–free conditions, the IEO system primarily generates vestibular-like epithelia rather than definitive cochlear fate. Therefore, the observed reduction in MYO7A- and SOX2-positive cells should be interpreted as a disruption of early otic sensory-epithelium formation rather than a cochlear-specific phenotype. The IEO defects reflect a general failure in prosensory differentiation, which does not contradict the preserved vestibular morphology observed in *Gdf6^delFull–/–^* mice.

### ATAC-Seq and epigenomic profiling reveal a dynamic enhancer landscape regulating GDF6 during otic differentiation.

**Figure 3:**
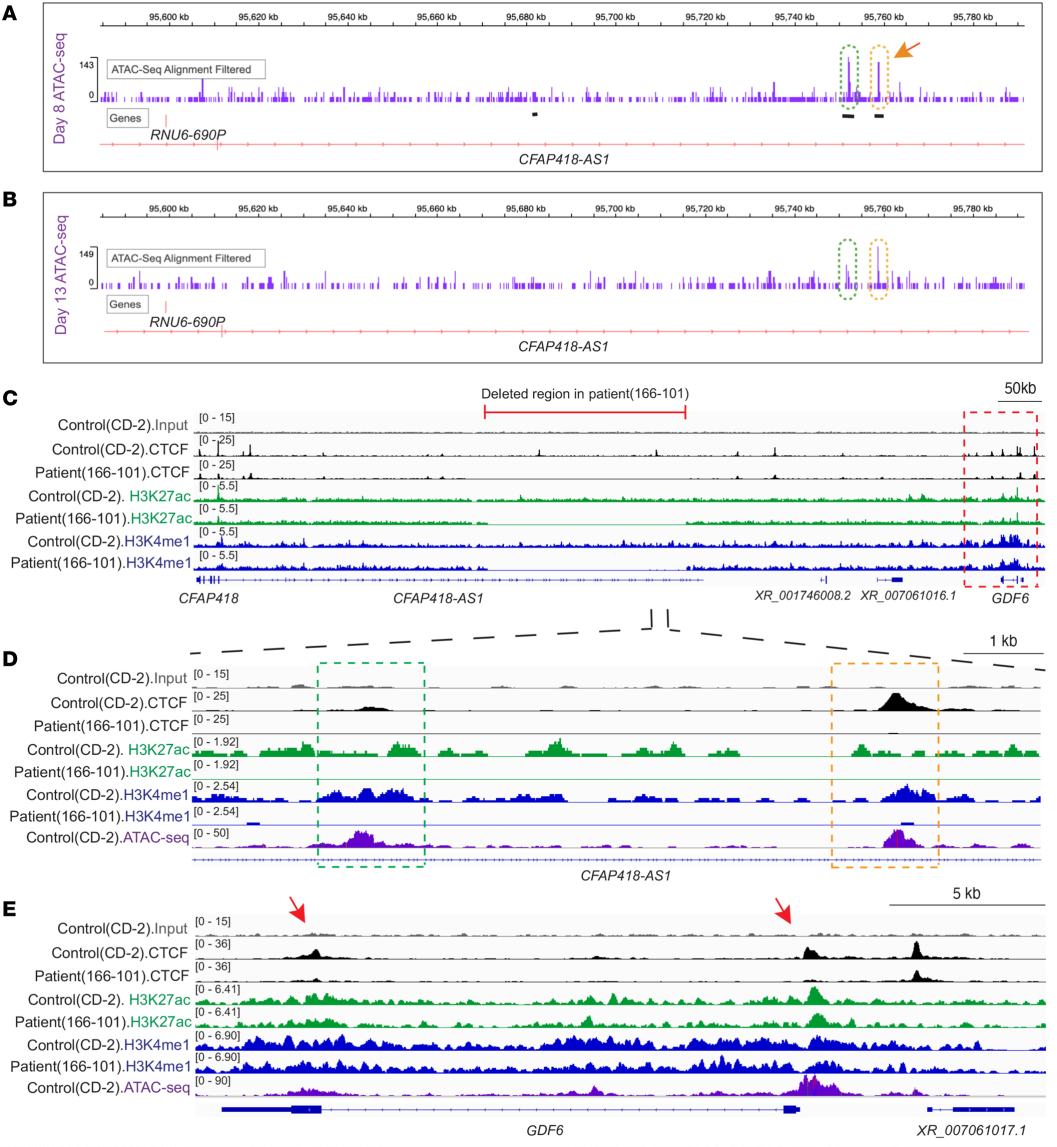
Chromatin accessibility and epigenomic profiling reveal CTCF binding sites and enhancer loss downstream of *GDF6* in cochlear aplasia. (**A** and **B**) ATAC-Seq profiles in control-derived inner ear organoids at day 8 (**A**) and day 13 (**B**) showcase dynamic open chromatin regions downstream of the *GDF6* locus (chr8: ~95.72–95.75 Mb, hg38), highlighted with dashed ovals. ATAC-Seq data are from 1 representative biological replicate at day 8 and day 13. The tracks are shown to illustrate relative accessibility patterns at 2 time points as indicated. The orange arrow indicates the previously identified region in scATAC-Seq in 2D otic progenitor model as shown in Supplemental Figure 7. Black bars under the peaks indicate the statistically significant peaks called in ATAC-Seq. (**C**) Normalized ChIP-Seq densities (CTCF, H3K27ac, and H3K4me1) across approximately 250 kb downstream of *GDF6* (chr8: 95,214,667–96,196,827) in control (CD-2) and patient (166-101) day 8 IEOs. The control shows strong enhancer signatures and CTCF binding at the candidate locus, which are absent in patient samples. The patient-derived deleted genomic region containing CTCF binding sites and *CFAP418-AS1* lncRNA is indicated with a red line; the *GDF6* locus is shown with dashed lines. The region shown was selected based on the pathogenic human deletions downstream of *GDF6* and is not derived from genome-wide peak selection. The absence of enhancer-associated histone marks and CTCF signal in patient tracks compared with WT reflects deletion of the underlying genomic region shown with a red line. (**D** and **E**) Normalized ChIP-Seq densities for CTCF, H3K27ac, and H3K4me1, as well as ATAC-Seq densities around CTCF binding sites/candidate enhancers in the patient-derived deleted genomic region (**D**) and at the *GDF6* locus (**E**) in patient IEOs compared with control at day 8. ATAC-Seq peaks overlapping with enhancer-associated marks and CTCF binding are shown with colored dashed lines in green and orange, respectively. CTCF binding sites are illustrated with red arrows. ChIP-Seq data are from 1 representative of 2 biological replicates for the control (CD-2) and 1 biological replicate for patient 166-101.

Previously characterized overlapping pathogenic deletions identified in 2 unrelated families with cochlear aplasia point to a common region affected (chr8:96,596,661–96,803,788; hg19), as reported by our group (1). Given the identified regulatory regions at early otic differentiation (Supplemental Figure 7, B and C), we aimed to determine chromatin accessibility in a *GDF6* locus–focused manner in the disease-linked regulatory interval using IEOs. To identify the regulatory regions responsible for *GDF6* activation during cochlear development, we performed ATAC-Seq on day 8 and day 13 IEOs derived from the control (CD2). Our analysis revealed distinct open chromatin regions located approximately 300–500 kb downstream of the *GDF6* locus (chr8q22.1; Figure 3, A and B), consistent with the regions shown by scATAC-Seq (Supplemental Figure 7C). These locus-specific sites remained accessible across early stages of otic differentiation on day 8 and day 13 IEOs, consistent with sustained regulatory activity during this developmental window. The broader window shown in Figure 3, A and B, provides regulatory context, and subsequent analyses refine the minimal critical region.

The disrupted CTCF sites reside within a large downstream gene desert, and *GDF6* is the only annotated gene within the same topologically associating domain. This topologically associating domain structure was previously reported in patients with overlapping pathogenic deletions causing cochlear aplasia (1). To further clarify the chromatin landscape, we performed ChIP-Seq for CTCF and the histone marks H3K27ac (active enhancer) and H3K4me1 (primed enhancer) in both control (CD2) and patient (166-101) iPSC-derived IEOs on day 8. The genome-wide comparison of chromatin profiles between control and patient-derived IEOs demonstrated reduced CTCF occupancy and altered enhancer-associated histone marks (Supplemental Figure 14). In control samples, we identified CTCF binding sites near H3K27ac and H3K4me1 peaks within the patient-deleted genomic region that contains the long noncoding RNA *CFAP418-AS1* (Figure 3C). Specifically, CTCF binding and the associated enhancer marks within the patient-deleted genomic region colocalized with ATAC-Seq peaks (Figure 3D). When examining the *GDF6* locus, we observed CTCF binding flanking *GDF6* alongside strong enrichment of H3K27ac and H3K4me1 in the same intergenic region, coinciding with ATAC-Seq peaks (Figure 3E). In addition, CTCF binding and enhancer mark localization along with ATAC-Seq peaks were observed at the *CFAP418-AS1* locus, and the reduction of CTCF binding and H3K27ac signal was noted in the patient compared with control samples (Supplemental Figure 15). However, the mouse ortholog of *CFAP418-AS1, 1700123O12Rik* RNA, was located outside of the critical region defined in the mouse model (Chr4:10,249,919–10,364,802) (Supplemental Figure 2). Importantly, the downstream regulatory interval containing enhancer-associated chromatin features and CTCF binding sites was deleted in patient-derived samples, resulting in the absence of ATAC-Seq and ChIP-Seq signals in this region. The arrangement of CTCF binding at the beginning and end of the *GDF6* gene suggests a possibly insulated gene structure with an active promoter (Figure 3E), and CTCF binding was reduced around the *GDF6* locus in the patient compared with control samples, although this function requires further investigation. In patient-derived IEOs, loss of CTCF binding and enhancer-associated marks (H3K27ac and H3K4me1) confined to the deleted intervals was consistent with the removal of this regulatory module. Notably, promoter-proximal chromatin features for the *GDF6* locus remained similar in the patient compared with control samples, suggesting that the loss of the genomic region in the patient sample disrupts the local regulatory region containing enhancer-CTCF binding. Because the deleted intergenic region in the patient sample lacked multiple epigenomic features, our analysis focused on identifying the CTCF-binding pattern and enhancer-associated marks at this region spanning the *GDF6* locus and their alteration in patient-derived IEOs. The disrupted regulatory regions containing enhancer-associated marks and CTCF binding correspond to the diminished *GDF6* expression and cochlear developmental failure.

Together, the loss of CTCF occupancy and depletion of H3K27ac/H3K4me1 specifically at the deleted enhancer region, accompanied by an approximately 50% reduction in *GDF6* expression in day 8 IEOs, supports the conclusion that this downstream interval contributes regulatory activity required for early *GDF6* activation in the early human otic lineage. These findings reflect disruption of a local enhancer-insulator unit rather than a locus-wide collapse of chromatin architecture. Given that patient-derived IEOs and the mouse deletion model interrogate distinct biological contexts, we interpret the in vitro molecular findings as complementary evidence rather than a direct phenocopy of the cochlear aplasia observed in *Gdf6^delFull–/–^* mice.

### CRISPRi targeting CTCF and enhancer region suppresses GDF6 expression, otic vescile formation and hair cell differentiation.

**Figure 4:**
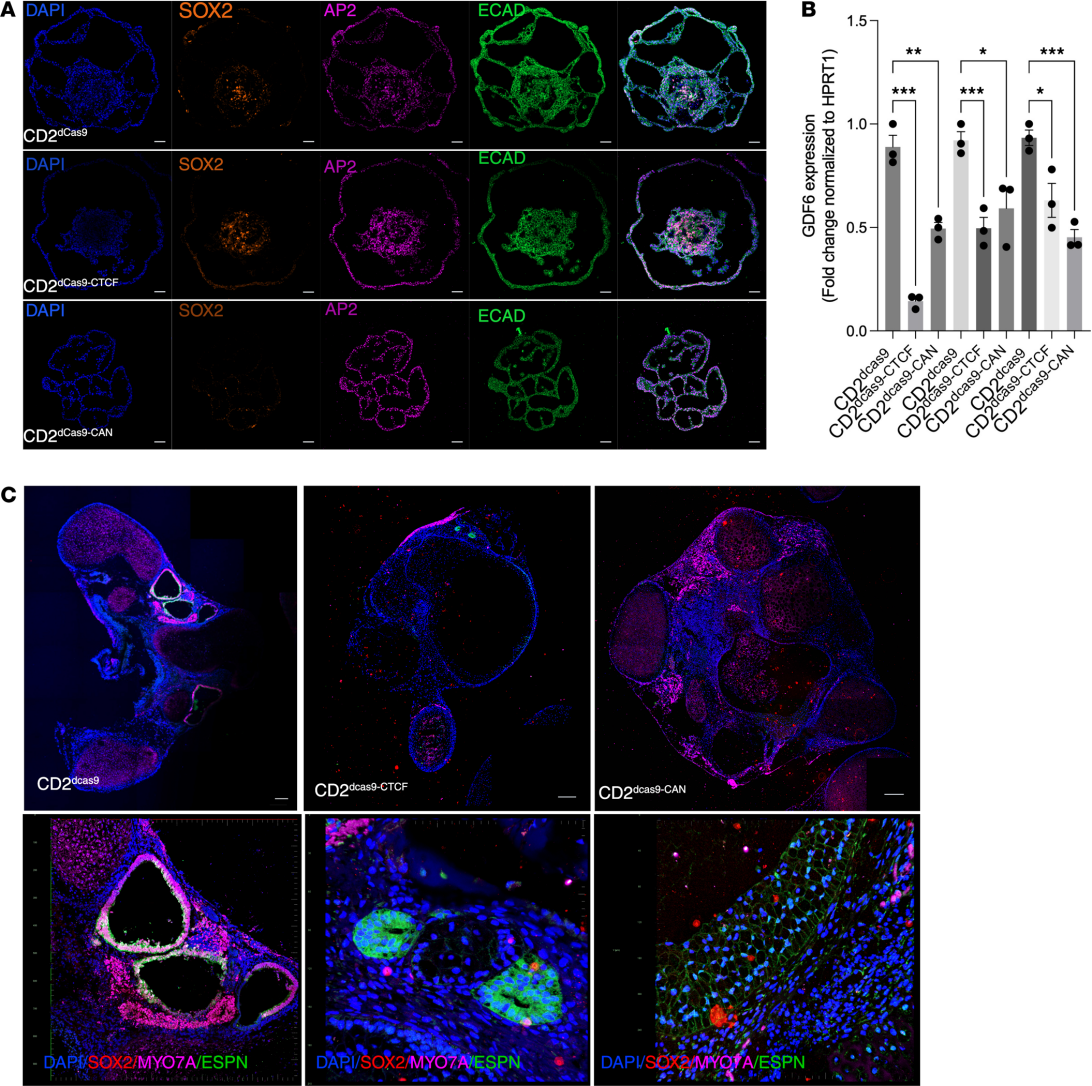
CRISPRi perturbation of a GDF6 regulatory region impairs otic epithelial organization and hair cell differentiation in IEOs. (**A**) Representative confocal immunofluorescence images (*n* = 3; 20× magnification; scale bar: 50 µm) of day 8 IEOs derived from parental CD2^dCas9^, CD2^dCas9-CTCF^ (targeting the putative CTCF site), and CD2^dCas9-CAN^ (targeting the candidate enhancer) lines, stained for DAPI (blue), SOX2 (orange), TFAP2A/AP2 (magenta), and CDH1/ECAD. (**B**) qRT-PCR analysis of GDF6 mRNA expression in IEOs at days 8, 12, and 15, normalized to *HPRT1*. Both CD2^dCas9-CTCF^ and CD2^dCas9-CAN^ lines show significantly reduced GDF6 expression compared with parental CD2^dCas9^ at all 3 time points (day 8: ***P < 0.001 for CTCF, **P < 0.01 for CAN; day 12: ***P < 0.001 for CTCF, *P < 0.05 for CAN; day 15: *P < 0.05 for CTCF, ***P < 0.001 for CAN). Data are presented as mean ± SEM from 3 biological replicates (each pooled from ~8 organoids) per sample. Statistical comparisons were performed by 1-way ANOVA followed by Tukey’s post hoc test for multiple comparisons. (**C**) Representative confocal immunocytochemistry of day 55 IEOs from CD2^dCas9^, CD2^dCas9-CTCF^, and CD2^dCas9-CAN^ lines stained for DAPI (blue), SOX2, MYO7A (magenta), and ESPN. The top row shows whole-organoid overviews (*n* = 2; 20× magnification); the bottom row shows high-magnification views (*n* = 2; 40× magnification; scale bar: 50 µm) of the same samples.

To further investigate the functional relevance of the CTCF and enhancer regions, CRISPRi was used to target a transcriptional repressor domain to these genomic loci. To that end, we generated CD2^dCas9-CTCF^ and CD2^dCas9-CAN^ targeting the CTCF (chr8:95,758,681–95,758,931; hg38) and enhancer regions (chr8:95,752,126–95,752,366; hg38), respectively (Supplemental Figure 8). The impact of the targeting of the transcriptional repressor to these loci on *GDF6* was assessed by qRT-PCR. By day 8, targeting either regulatory domain disrupted the regular compartmentalization of IEOs, resulting in disorganized CDH1-positive epithelia (Figure 4A). This phenotype was accompanied by reduced *GDF6* expression following perturbation of both domains, with suppression evident at day 8 and maintained through day 15 (Figure 4B). CRISPRi targeting of distal regulatory intervals, including the CTCF-associated region and the candidate enhancer region, reduced *GDF6* expression and disrupted early sensory-epithelium organization, supporting a role for these loci in proper regulation of *GDF6*.

By day 55, control (CD2^dCas9^) IEOs formed cavities with discrete hair cells (Espin [ESPIN]^+^/MYO7A^+^) and supporting cells (SOX2^+^) (Figure 4C). At the same time, the CRISPRi-perturbed IEOs showed scattered, mislocalized SOX2^+^ cells and cavities lacking a coherent sensory epithelium (Figure 4C and Supplemental Figure 20), imitating the findings from patient-derived IEOs. Additional single-channel confocal imaging further confirmed the disrupted organization of hair cell–like and supporting cell markers in CRISPRi-perturbed IEOs (Supplemental Figure 20). Similar expression differences have also been seen at the mRNA level for *ATOH1*, *SOX2,* and *BRN3c* (Supplemental Figure 21). Collectively, these findings indicate that early disruption of these loci leads to *GDF6* depletion and impaired otic lineage specification, resulting in IEOs with a marked loss of organized sensory-epithelium–like domains. Given the vestibular bias of the current sonic hedgehog–free protocol, we interpret these changes as modeling early otic sensory-epithelium failure rather than a direct in vitro phenocopy of cochlear aplasia because both the vestibule and the cochlea develop from the otic-epibranchial progenitor domain during embryonic development.

## Discussion

Our previous identification of a homozygous, approximately 200 kb deletion located approximately 350 kb downstream of *GDF6* in patients with cochlear aplasia implicates *GDF6* as a key factor in early cochlear development controlled by distal enhancers (1, 9). Here, we narrow that region to approximately 100 kb using *Gdf6^delFull–/–^* and *Gdf6^del3′–/–^* mice generated in this study. Although monoallelic *GDF6* mutations in humans cause skeletal and ocular defects (22, 23), the phenotype observed here suggests that disruption of otic-lineage enhancer activity has a more marked effect on cochlear development than the partial loss of function from coding mutations. The deleted interval functions as an enhancer-rich regulatory hub for *GDF6*. Importantly, no other protein-coding genes are located within this insulated domain, supporting the specificity of the regulatory effects observed here. This genome architecture explains why chromatin accessibility changes and transcriptional consequences are restricted to *GDF6* (1). This 3D genomic organization likely facilitates specific enhancer-promoter interactions required for developmental maintenance of *GDF6* in the cochlea. To functionally test the contribution of the downstream regulatory elements, we used CRISPRi to repress either the CTCF site or the adjacent enhancer region. The presence of a CTCF site and nearby enhancer element within the deleted interval was identified through CTCF ChIP-Seq and enhancer-associated histone-mark profiling. CRISPRi targeting of the distal regulatory interval overlapping the CTCF-associated region reduced *GDF6* expression, supporting a role for this locus in proper regulation of *GDF6*. Whether loss of this locus also reorganizes chromatin architecture or enhancer-promoter contacts remains to be explored in future chromosome conformation capture assays. Given the context-dependent roles of CTCF as a multifunctional protein, we interpret these results as loss of a composite regulatory element containing CTCF binding and adjacent enhancer marks. Our findings support a model in which the deleted downstream interval harbors a possible developmental maintenance enhancer that stabilizes *GDF6* expression during otic epithelial maturation.

Although we did not directly assay chromatin looping, the dependence of *GDF6* expression on this enhancer-containing region is consistent with a regulatory architecture that supports sustained enhancer-promoter communication during cochlear development. The enhancer site we identified in this study is proximal to the CTCF site, a characteristic feature of super-enhancers that often sit adjacent to or are bracketed by CTCF-cohesin loop anchors (19, 24). It is important to note that scATAC-Seq provided the initial set of regulatory regions at the *GDF6* locus in patient- versus control-derived otic progenitor cells. Afterward, bulk ATAC-Seq accessibility was used to verify and refine the identified region in control-derived IEOs; therefore, chromatin accessibility changes in patient IEOs could not be inferred. Because the downstream enhancer interval is deleted in patient samples, our conclusions focus on the absence of the regulatory sequence itself, rather than on a quantitative comparison of altered enhancer or CTCF binding between patient and control samples. The patient IEO phenotype (Figure 2) and the CRISPRi perturbation (Figure 4) converge on a single model: loss of GDF6 regulatory control first disrupts otic epithelial organization (reduced CDH1, PAX2), and this early defect precedes the loss of MYO7A^+^ hair cell–like cells. The 2 experiments probe the same axis, GDF6-dependent sensory epithelium development, from different directions.

The disorder described here is an enhanceropathy, a condition caused by loss of tissue-specific enhancer function (11). In this case, loss of a noncoding regulatory domain produces a developmental anomaly indistinguishable from a coding loss of function. Disruption of 3D genome organization is an increasingly recognized cause of congenital disease (25). Structural variants such as deletions or inversions can rewire enhancer-promoter interactions, causing gene silencing (enhancer disconnection) or ectopic activation (enhancer adoption) (25). In silico estimates suggest that 10%–30% of congenital anomalies in patients with structural variants result from such long-range regulatory disruptions (26–28). In this study, removing the *GDF6* enhancer domain phenocopies a gene KO, demonstrating that congenital hearing loss can arise from disrupted genome architecture rather than a coding mutation.

Because current IEO differentiation protocols lack sonic hedgehog signaling, the IEOs mainly develop vestibular-like identities. As a result, the deficits observed in patient-derived IEOs reflect impaired early otic sensory-epithelium development rather than a direct in vitro analog of cochlear aplasia. This distinction clarifies why hair-cell and supporting-cell loss occurs in vitro while vestibular structures are preserved in *Gdf6^delFull–/–^* and *Gdf6^–/–^* mice. These differences between the organoid and in vivo phenotypes indicate that the organoid model captures key aspects of the developmental defect but does not fully recapitulate the in vivo phenotype.

CTCF is a multifunctional regulatory protein whose effects on gene expression are highly context dependent. Although we did not directly assess chromatin looping or topologically associating domain architecture, our data indicate that loss or repression of disease-associated CTCF binding sites disrupts *GDF6* transcription, supporting a functional role for CTCF in maintaining appropriate regulatory interactions at this locus. Chromosome conformation capture studies such as genome-wide (high-throughput) chromosome conformation capture, micrococcal nuclease-based chromosome conformation capture, or locus specific circularized chromosome conformation capture followed by sequencing, combined with targeted base editing of the CTCF motif itself, will be needed to resolve whether this module acts through a fixed loop or through dynamic enhancer-promoter contacts.

## Methods

### Animals

#### Sex as a biological variable.

This study included both human samples and animal models, but it was not specifically designed to identify sex-dependent differences, so sex was not treated as a biological variable in the statistical analysis. No consistent effects based on sex were observed across the reported experimental outcomes.

#### Generation of cis-regulatory deletion mouse models using CRISPR/Cas9.

To generate *Gdf6^delFull–/–^* and *Gdf6^del3^′^–/–^* mouse models, which feature deletions of approximately 221 kb (Chr4:10,249,919–10,471,132, mm10) and approximately 106 kb (Chr4:10,364,802–10,471,054, mm10), respectively, CRISPR/Cas9 genome editing was performed in mice with a C57BL/6J genetic background (29). Six sgRNAs were designed using CRISPOR (30) to flank the targeted deletion regions, with 2 sgRNAs positioned near the 5′ boundary of each deletion and 2 shared sgRNAs at the 3′ end. Cas9 protein and crRNAs (Integrated DNA Technologies, Inc.) were ordered. crRNAs and tracrRNA (Alt-R CRISPR guide RNAs, Integrated DNA Technologies, Inc.) were annealed in a thermocycler to make active sgRNA. The sgRNA was then mixed with Cas9 protein to form ribonucleoprotein (RNP) complexes and was microinjected into one-cell-stage C57BL/6J mouse zygotes, following established protocols (29, 31). Complete sgRNA sequences are available in Supplemental Table 1.

After microinjection of the RNP complexes, 373 zygotes were selected, and 370 embryos were transferred into the oviducts of pseudo-pregnant recipient mice, resulting in 48 live pups. Founder (F0) mice were screened for the expected deletions using PCR and sequencing. Initial PCR screening identified 7 mice with potential deletions, and sequencing confirmed deletions in 5 of these animals. Founder lines 30 and 31 had the 106 kb deletion; lines 11, 23, and 25 possessed the 221 kb deletion. To establish stable deletion lines, founder 30 (106 kb deletion) and founder 23 (221 kb deletion) were bred to WT C57BL/6J mice (The Jackson Laboratory, Jax stock 000664), producing F1 heterozygous carriers. These heterozygotes were intercrossed to generate homozygous deletion mutants, which were validated using junction PCR.

Briefly, the expected amplicon sizes for the WT allele, *Gdf6^del3′–/–^* and *Gdf6^delFull–/–^* were 620 bp, 1,100 bp, and 700 bp, respectively. Specific primers were used to genotype each deletion. The 106 kb deletion was detected using the forward primer 5′-AAAGGCCACAGAGATCACTTG-3′ and the reverse primer 5′-CAAAGACTTTCCCTCCTGAGG-3′, yielding a PCR product of approximately 1 kb. For the 221 kb deletion, the forward primer 5′-AAGACCAACACACCCTGTCC-3′ and the same reverse primer 5′-CAAAGACTTTCCCTCCTGAGG-3′ were employed, producing a PCR product of approximately 0.9 kb. PCR amplification was performed using Go-Taq Hot Start Green Mix (Promega) under the following conditions: an initial denaturation step at 95°C for 2 minutes, followed by 40 cycles of 95°C for 20 seconds, 62°C for 20 seconds, and 72°C for 45 seconds, with a final extension at 72°C for 5 minutes. The CRISPR targeting strategy, sgRNA locations, and deletion sizes are illustrated in Figure 1A and Supplemental Figures 2–5. Mouse transgenesis, embryo transfer, and animal husbandry were performed according to standard procedures (20). All the mouse CRISPR/Cas9 editing procedures were done at the Mouse Genome Engineering Core Facility, University of Nebraska Medical Center, Omaha, Nebraska, USA. The mouse colony was then expanded at the University of Miami, and the resulting animals were housed 2–5 per cage in a room with a 12-hour light/12-hour dark cycle (lights on at 6 am, off at 6 pm) with ad libitum access to food and water. All animal procedures were performed under a protocol approved by the IACUC at the University of Miami.

#### Auditory brainstem response evaluation.

Auditory brainstem responses (ABRs) were conducted using a Smart EP Universal Smart Box (Intelligent Hearing Systems). The ABR stimuli included 0.1 ms duration clicks and pure-tone pips at 8, 16, and 24 kHz. Click stimuli were enveloped in a rectangular window while a Blackman window surrounded pips. The stimuli were presented at 20 dB sound pressure level (SPL) and increased in 10 dB steps to 100 dB SPL. A total of 600 sweeps were averaged for each frequency and amplitude. ABR thresholds were determined for each stimulus frequency by identifying the lowest intensity that produced a recognizable ABR pattern defined as having at least 2 consistent peaks above the baseline.

#### Inner ear clearing for cochlear morphology.

To determine whether mutant mice exhibited morphological changes in the inner ear, specimens from WT, *Gdf6^delFull–/–^,* and *Gdf6^del3′–/–^* mice at 6 months of age were harvested and fixed overnight in Bodian fixative. The organs were then placed in 70% ethanol with agitation for 24 hours and subsequently transferred to 3% KOH, refreshed daily, until all soft tissue was completely dissolved. The samples were cleared in a solution of glycerol, 70% ethanol, and benzyl alcohol in a 2:2:1 ratio for approximately 24 hours. Finally, the samples were preserved in a 1:1 mixture of glycerol and 70% ethanol for further analysis.

#### RT-PCR and qRT-PCR for Gdf6 expression in mice.

To examine the expression of *Gdf6* in various tissues, the brain, inner ear, eye, limbs, and heart were dissected from 17-day post-coitum embryos of WT and *Gdf6^delFull–/–^* mice. Following the manufacturer’s instructions, total RNA was isolated using an RNeasy Mini kit (QIAGEN). Before reverse transcription, RNA samples were treated with DNase I (QIAGEN). cDNA was synthesized using a high-capacity RNA-to-cDNA kit (Applied Biosciences). *Gdf6* mRNA expression in WT and *Gdf6^delFull–/–^* mice was evaluated. RT-PCR reactions were performed using the forward primer 5′-TTACTCCATTGCCGAGAAGC-3′ and the reverse primer 5′-GGCGATAAAGCCTTAGCTCTG-3′ to amplify a 200 bp fragment of the *Gdf6* transcript. For *Gapdh,* a 129 bp fragment was amplified using the forward primer 5′-AGGTCGGTGTGAAC-3′ and the reverse primer 5′-TGTAGACCATGTAGTT-3′. qRT-PCR was performed using SYBR Green Master Mix (Applied Biosystems). Data were collected using the QuantStudio 6 Flex Real-Time PCR System (Applied Biosystems/Invitrogen). Data analysis was conducted to determine gene fold-changes using the comparative cycle threshold (ΔΔCt) method, relative to the housekeeping gene *Gapdh*.

### IPSC derivation and karyotyping

We derived iPSCs from dermal fibroblasts obtained from both the proband (166-101) carrying approximately 220 kb noncoding deletion downstream of *GDF6* (Chr8:96,596,661–96,803,788; hg19) and the controls from healthy donors (CA-26 and CD2), as previously described (1). Following the manufacturer’s protocol, fibroblasts were treated with the CytoTune iPS 2.0 Sendai Reprogramming kit (Thermo Fisher Scientific). After 7 days, the cells were transferred from Matrigel-coated culture plates to E8-Flex media. Emerging iPSC colonies were manually selected based on their morphology and the expression of the pluripotency marker TRA-1-60. Clonally derived lines were expanded, and individual clones were validated for pluripotency using immunocytochemistry for the pluripotency markers OCT4, SOX2, and TRA-1-60.

Additionally, G-band karyotyping was performed to confirm the absence of gross chromosomal abnormalities during reprogramming and expansion (Supplemental Figure 6). All iPSC derivation and validation procedures were carried out at the Stem Cell Core Facility of the Hussman Institute for Human Genomics, University of Miami.

### Early otic lineage cell differentiation

We adapted the differentiation protocol to generate early-otic-lineage progenitor cells, as previously described (1, 32). iPSCs were plated at 10,000 cells/cm² density on vitronectin-coated plates (Life Technologies) and cultured in mTESR1 medium (Stemcell Technologies). On day 3, the medium was replaced with N2B27-CDM composed of DMEM/F12 (1:1 containing 1× N-2 supplement; Thermo Fisher Scientific), 1× B-27 supplement (Thermo Fisher Scientific), 1× nonessential amino acids (Thermo Fisher Scientific), 1× GlutaMAX (Thermo Fisher Scientific), and 100 μM β-mercaptoethanol, further supplemented with 10 ng/mL FGF2 (Peprotech), 10 ng/mL BMP4 (Peprotech), and 1 μM SB431542 (Stemcell Technologies). By day 6, the culture conditions were adjusted to promote pre-placodal ectoderm (27) differentiation by adding 10 ng/mL FGF2, 1 μM SB431542, 2 μM IWP-2 (a WNT inhibitor), and 100 nM LDN193189 (a BMP inhibitor). On day 11, cells were dissociated into a single-cell suspension using Accutase, followed by negative selection for TRA-1-81 (a pluripotency marker) and positive selection for CD271 (a marker for early otic progenitor cells).

### scATAC-Seq and scRNA-Seq library preparation and sequencing

iPSCs were differentiated into early otic lineage cells as described above. After dissociation of cells with Accutase followed by negative selection for TRA-1-81 and positive selection for p75 using CD271 magnetic beads (Miltenyi Biotec), the cells were used for single-cell analysis. This CD271-enriched population was utilized for single-nuclei isolation and processed through the 10X Genomics multiome pipeline for both scATAC-Seq and gene expression profiling, as per user specifications. Single-cell library preparation was conducted using Chromium X with sequencing performed at the Center for Genome Technology DNA Sequencing Core, John P. Hussman Institute for Human Genomics (Supplemental Figure 7). The sequencing data were aligned with CellRanger and analyzed using Seurat/Signac (33, 34). ArchR was used to compare the scATAC-Seq peaks in patient versus control samples at the locus-specific region as shown in Supplemental Figure 7C (35).

### Generation of CRISPRi CD2dCas9 line

To generate the CRISPRi CD2-dCas9 cell line, CD2 cells were transduced with hCMV-Blast-dCas9-SALL1-SDS3 lentiviral particles (Horizon Discovery) at a low MOI (0.3) to ensure single-copy integration per cell. Briefly, CD2 cells were plated at 50% confluency and transduced in the presence of 4 μg/mL polybrene (Sigma-Aldrich) to enhance infection efficiency. After 24 hours, the medium was replaced, and the cells were allowed to recover for 72 hours. Subsequently, the cells were grown for 15 days under blasticidin selection (2 μg/mL, Thermo Fisher Scientific). Monoclonal colonies were isolated using cloning cylinders (Sigma-Aldrich) and expanded in blasticidin-containing medium. Immunocytochemistry was used to validate clonal cell lines for stable dCas9 expression and pluripotency (Supplemental Figure 18).

### CRISPRi guide RNA cloning, lentiviral production, and generation of CD2dcas9-CTCF and CD2dcas9-CAN monoclonal lines

CRISPRi sgRNAs were designed to target the identified CTCF sites (chr8:95,758,681–95,758,931) and a candidate regulatory locus (chr8:95,752,126–95,752,366) using Benchling (https://benchling.com). These guide RNAs were cloned into the lentiGuide-Puro and lentiGuide-Neo lentiviral backbones via BsmBI digestion (Supplemental Figure 17), following previously described methods (36). The lentiGuide-Puro backbone was a gift from Feng Zhang (Broad Institute of MIT and Harvard, Cambridge, Massachusetts, USA; Addgene plasmid 52963), and the lentiGuide-Neo backbone was a gift from Caroline Goujon (Institut de Recherche en Infectiologie de Montpellier, Montpellier, France; Addgene plasmid 139449). Information on the sgRNAs is provided in Supplemental Table 2, and detailed schematics of gRNA placement are depicted in Supplemental Figure 8.

Lentiviral particles were generated using Lenti-X Packaging Single Shots (Takara Bio) according to the manufacturer’s protocol. Viral titers were assessed using Lenti-X GoStix Plus (Takara Bio). CD2^dcas9^ iPSC cells underwent overnight transduction with 4 μg/mL polybrene (Sigma-Aldrich). After transduction, the cells were allowed to recover for 72 hours before undergoing sequential dual antibiotic selection: first with 0.2 μg/mL puromycin for 14 days, followed by 150 μg/mL geneticin (G418) for an additional 14 days to establish CD2^dcas9^-^CTCF^ and CD2^dcas9^-^CAN^ lines. Consequently, the resulting CD2^dcas9-CTCF^ and CD2^dcas9-CAN^ lines were expanded and validated for pluripotency maintenance (Supplemental Figure 19).

### Generation of IEOs

IEOs were generated using CD2^WT^ and patient (166-101) or CD2^dcas9^, CD2^dcas9-CTCF^, and CD2^dcas9-CAN^ lines, following the established protocols of Koehler et al. (37) and the modifications of Valk et al. (38). iPSCs were cultured on vitronectin-coated plates in E8-Flex medium, supplemented with 100 μg/mL Normocin (InvivoGen). On day –2 of differentiation, iPSCs were dissociated using StemPro Accutase (Thermo Fisher Scientific) and filtered through 70 μm strainers. A total of 3,500 cells per well were seeded in 96-well Nunclon Sphera U-bottom plates (Thermo Fisher Scientific), with 100 μL of E8-Flex medium containing 20 μM Y-27632 (Stemcell Technologies). The following day, an additional 100 μL of fresh E8-Flex medium was added, reducing the final concentration of Y-27632 to 10 μM.

On day 0, the aggregates were collected and washed 3 times with E6 medium (Life Technologies) containing 100 μg/mL Normocin. They were then transferred to new 96-well U-bottom plates with 100 μL of E6 medium supplemented with 2% GFR-Matrigel (Corning), 10 μM SB431542 (Stemcell Technologies), 4 ng/mL FGF2 (Peprotech), and 2.5 ng/mL BMP4 (Peprotech).

On day 3, 25 μL of E6 medium containing 100 μg/mL Normocin, 1 μM LDN193189 (Stemgent, Reprocell), and 250 ng/mL FGF2 was added, increasing the total volume to 125 μL. On day 6, an additional 75 μL of E6 medium with 100 μg/mL Normocin was added.

Otic vesicle induction began on day 8 by adding E6-medium supplemented with 100 μg/mL Normocin and 3 μM CHIR99021. On day 10, the medium was replenished to maintain CHIR99021 levels.

On day 12, aggregates were washed 3 times and transferred to 24-well low-attachment plates (Thermo Fisher Scientific) in 500 μL of IEO maturation medium (32) composed of Advanced DMEM/F12, Neurobasal medium, 1× GlutaMAX, 0.5× B-27 supplement minus vitamin A, 0.5× N-2 supplement, 0.1 mM 2-mercaptoethanol, 100 μg/mL Normocin, 1% GFR-Matrigel, and 3 μM CHIR99021. Organoids were maintained on an orbital shaker at 65 rpm in an incubator set at 37°C with 5% CO_2_.

On day 15, half of the medium was replaced with organoid maturation medium (OMM) containing 1% GFR-Matrigel and 3 μM CHIR99021. On day 18, half of the medium was replaced with OMM without Matrigel or CHIR99021. From day 18 onward, the aggregates were maintained with half-medium changes every third day and a complete medium change every sixth day until maturation.

### RNA isolation, cDNA synthesis, and TaqMan assay

IEOs were washed twice with PBS for RNA extraction on days 8, 12, and 15, and RNA was isolated using TRIzol reagent (Sigma-Aldrich) according to the manufacturer’s instructions. Briefly, IEOs were homogenized by intermittent vortexing in 1 mL of TRIzol, followed by the addition of 300 μL of chloroform. The homogenate was separated into 3 phases: an upper aqueous layer (containing RNA), an interphase, and a lower organic layer (comprising DNA and proteins). RNA was precipitated from the aqueous phase with 500 μL of isopropanol, washed twice with 70% ethanol, and quantified. cDNA synthesis was performed using qScript XLT cDNA SuperMix (Quantabio) according to the manufacturer’s protocol. To analyze *GDF6* mRNA expression, cDNA from both proband and control samples was subjected to qRT-PCR using a 6-carboxyfluorescein (FAM)-conjugated *GDF6* TaqMan probe (HS01377663_m1) and a VIC-conjugated (Applied Biosystems) *HPRT* probe (HS02800695_m1) as a control. The reaction was set up using TaqMan Gene Expression Master Mix (Thermo Fisher Scientific) and run on a QuantStudio 6 Flex Real-Time PCR System. The results were expressed as a fold-change normalized to *GAPDH*.

### Processing of IEOs and immunocytochemistry

Mature IEOs were washed twice with PBS and fixed in 4% paraformaldehyde (PFA) at 4°C. After fixation, the IEOs were washed 3 times with PBS and processed at the Cancer Modeling Shared Resource (CMSR) at Sylvester Cancer Center, University of Miami. Briefly, fixed IEOs were embedded in gelatin, serially dehydrated through graded ethanol, and embedded in paraffin wax. Sections were cut at 8 μm thickness. Before staining, paraffin was removed with xylene and sections were rehydrated through a descending ethanol series. Antigen retrieval was performed by heat-induced epitope retrieval in citrate buffer (pH 6.0). Sections were then permeabilized with 0.2% Triton X-100 in PBS for 15 minutes and blocked using High Protein Blocking buffer (Invitrogen, 00-4952-54). For cryosectioning (Figure 2G), IEOs were cryoprotected by sequential incubation in 15% and 30% sucrose for 12 hours, embedded in Tissue-Tek OCT (Sakura), and sectioned at 12 μm using a Leica cryostat. Before staining, slides were brought to room temperature, washed 5 times with PBS to remove residual OCT, permeabilized with 0.3% Triton X-100 in PBS for 10 minutes, and blocked for 1 hour at room temperature in 5% BSA and 0.01% Tween 20 in PBS. For both methods, primary antibody incubation was performed overnight at 4°C in a humidified chamber, using antibodies against MYO7A (Myosin VIIa), SOX2, and ESPN (unless otherwise stated in the figure legend). The following day, sections were washed 3 times with PBS and stained with secondary antibodies for 2 hours at room temperature. Slides were washed 5 times with PBS, mounted in anti-fade mounting medium with DAPI (Abcam), and imaged using an LSM710 confocal microscope (Zeiss).

### ATAC-Seq library preparation and sequencing

ATAC-Seq libraries were prepared and sequenced by Azenta Life Sciences using the Illumina 2 × 150 bp paired-end configuration. Chromatin was isolated from pooled material derived from approximately 200 day 8 IEOs to meet assay input requirements; individual IEOs do not yield sufficient material for ATAC-Seq library preparation. Sample quality and quantity were assessed before sequencing to ensure optimal performance. Sequencing data were processed through a standardized bioinformatics pipeline that included quality control, alignment, filtering, deduplication, peak calling, and differential analysis. Raw sequencing reads were assessed for quality using FastQC, followed by adapter trimming and removal of low-quality bases using Trimmomatic (v0.38) (39). Cleaned reads were aligned to the hg38 reference genome with Bowtie2 using default parameters. Sequencing statistics were calculated for each sample, including total reads, yield, and base quality. Postprocessing involved filtering aligned reads with Samtools (v1.9) (40) to retain only primary alignments, applying a minimum mapping quality of 30. Picard (v2.18.26) was used to mark and remove PCR and optical duplicates, as well as reads mapping to mitochondrial DNA; unplaced contigs were discarded prior to peak calling. Open chromatin regions were identified using MACS2 (v2.1.2) (41), excluding blacklisted genomic regions to minimize false-positives. Peaks detected in at least 66% of samples for a given condition were retained for downstream analysis. Processed data, including raw FASTQ files, aligned BAM files, and peak-called BED files, were delivered to the investigators. Final deliverables included raw sequencing reads in FASTQ format, aligned reads in BAM format, and processed peak files for each sample and condition. This workflow ensures the generation of high-quality ATAC-Seq data suitable for open chromatin and regulatory element analysis.

### ChIP-Seq and analysis

ChIP-Seq experiments were conducted as previously described (42, 43). In brief, approximately 200 pooled IEOs were dissociated using trypsin; the process was halted by the application of whole media. After washing in 1× PBS, the cells were fixed with 2 mM disuccinimidyl glutarate (DSG) for 30 minutes at room temperature, and then cross-linked with 1% formaldehyde. Nuclei were isolated, and chromatin was fragmented to approximately 250 bp using a Covaris E220. ChIP was performed using antibodies against CTCF, H3K27ac, and H3K4me1. Chromatin from *Drosophila* (at a 1:100 ratio to IEO-derived chromatin) and the *Drosophila*-specific H2Av antibody were utilized as a spike-in control in each sample. For ChIP-Seq, libraries were prepared using 1–30 ng of immunoprecipitated DNA (44).

ChIP-Seq data were analyzed as before (42, 44). Briefly, sequence reads were mapped to the hg38 or dm6 reference genomes using Bowtie 2 (version 2.3.4.1) with default parameters (43). Quality filtering and duplicate removal were performed using SAMtools (v1.9) (40). MACS (v1.4.2) was used for narrow peak calling with the default parameter of “macs2” (41). After normalization with total read counts, ChIP-Seq heatmaps, density plots, and tracks were generated. Heatmaps were generated using DeepTools in R (41). The ChIPpeakAnno package (v3.36.1) from Bioconductor (45) was utilized to generate overlap among ChIP-Seq samples in Venn diagrams. The sizes of the Venn diagrams were drawn using an online tool (http://barc.wi.mit.edu/tools/venn/). Normalized ChIP-Seq read densities were visualized in the Integrative Genomics Viewer (46).

### Antibodies

The following primary antibodies were used for immunofluorescence staining at a dilution of 1:100/CTCF (Abcam, ab128873), H3K27ac (Abcam, ab4729), H3K4me1 (Abcam, ab8895), *Drosophila*-specific H2Av antibody (Active Motif, 61751), anti-E-cadherin (CDH1) rabbit monoclonal (clone 24E10, Cell Signaling Technology, 3195), anti-E-cadherin mouse monoclonal (clone 4A2, Cell Signaling Technology, 14472), anti-GDF6 rabbit polyclonal (Abcam, ab73288), anti-phospho-SMAD1/5/9 rabbit monoclonal (clone D5B10, Cell Signaling Technology, 13820), anti-MYO7A mouse monoclonal (clone 138-1, Developmental Studies Hybridoma Bank), anti-ESPN rabbit polyclonal (Abnova, PAB22204), anti-AP-2α mouse monoclonal (clone 3B5, Developmental Studies Hybridoma Bank), anti-SOX2 rat monoclonal (eBioscience/Invitrogen, 14-9811-80), anti-PAX2 rabbit polyclonal (Invitrogen, 71-6000), anti-PAX8 rabbit polyclonal (Proteintech, 10336-1-AP), anti-NANOG rabbit monoclonal (Cell Signaling Technology, 8822), anti-OCT4A rabbit monoclonal (Cell Signaling Technology, 2840), and anti-TRA-1-60 mouse monoclonal (Cell Signaling Technology, 4746). Secondary antibodies, all raised in goat and used at 1:500, included Alexa Fluor 488–conjugated anti-rabbit IgG (Invitrogen, A11008), Alexa Fluor 488–conjugated anti-rat IgG (Invitrogen, A32790), Alexa Fluor 594–conjugated anti-rat IgG (Invitrogen, A21209), Alexa Fluor 594–conjugated anti-mouse IgG (Invitrogen, A21235), and Alexa Fluor 647–conjugated anti-mouse IgG (Invitrogen, A31527). Coverslips were mounted using ProLong Gold Antifade Mountant (Thermo Fisher Scientific, P36985).

### Statistics

Statistical analyses were performed using 1-way or 2-way ANOVA as appropriate, followed by Tukey’s post hoc test for multiple group comparisons. For pairwise comparisons, independent 2-tailed *t* tests were used to assess statistical significance. Data are presented as mean ± SEM, with *P* less than 0.05 deemed statistically significant. All analyses were conducted using GraphPad Prism (v10).

### Study approval

Animal experiments were reviewed and approved by the IACUC at the University of Miami and carried out in accordance with the NIH guidelines “Using Animals in Intramural Research” (https://policymanual.nih.gov/3040-2?). Studies involving human participants were approved by the Ethics Committee of Ankara University, Ankara, Turkey (protocol 012413) and the IRB at the University of Miami (protocol 20081138). Written informed consent was obtained from each participant; for minors, consent was provided by a parent or legal guardian.

### Data availability

The sequencing data have been deposited in NCBI’s Gene Expression Omnibus (GEO) under superseries GSE329999, which comprises GSE328473 (ChIP-Seq) and GSE329998 (ATAC-Seq). The 10x Multiome data are available under GSE331301. Any additional data or materials can be obtained from the corresponding author upon reasonable request. Human-derived iPSC lines are subject to institutional material transfer agreements due to patient consent restrictions. The Supporting data values are available in the supplemental Excel file.

## Author contributions

MFZ, CA, and HO conducted experiments, acquired and analyzed data, and wrote the manuscript. MR conducted experiments and acquired data. DMD, JIY, GB, and DD provided reagents, designed research studies, and wrote the manuscript. MCR, RMQ, KW, and CG designed research studies, conducted experiments, and acquired and analyzed data. SG and AG analyzed data. MT designed research studies, conducted experiments, acquired and analyzed data, provided funding and reagents, and wrote the manuscript.

## Conflict of interest

The authors have declared that no conflict of interest exists.

## Funding support

This work is the result of NIH funding, in whole or in part, and is subject to the NIH Public Access Policy. Through acceptance of this federal funding, the NIH has been given a right to make the work publicly available in PubMed Central.

- NIH grants R01DC009645 and R01DC012836 (to MT).

## Supplementary Material

Supplemental data

Supporting data values

## Version 1 06/18/2026

In-Press Preview

## Version 2 08/10/2026

Electronic publication
