# Supplementary material for: Distal enhancer-insulator module of GDF6 is essential for cochlear formation
Source: JCI Insight. 2026 Jun 18;11(15):e195462. doi: 10.1172/jci.insight.195462 (PMC13463603; doi:10.1172/jci.insight.195462)
Supplement: Supplementary file 1 [file jciinsight-11-195462-s106.pdf]

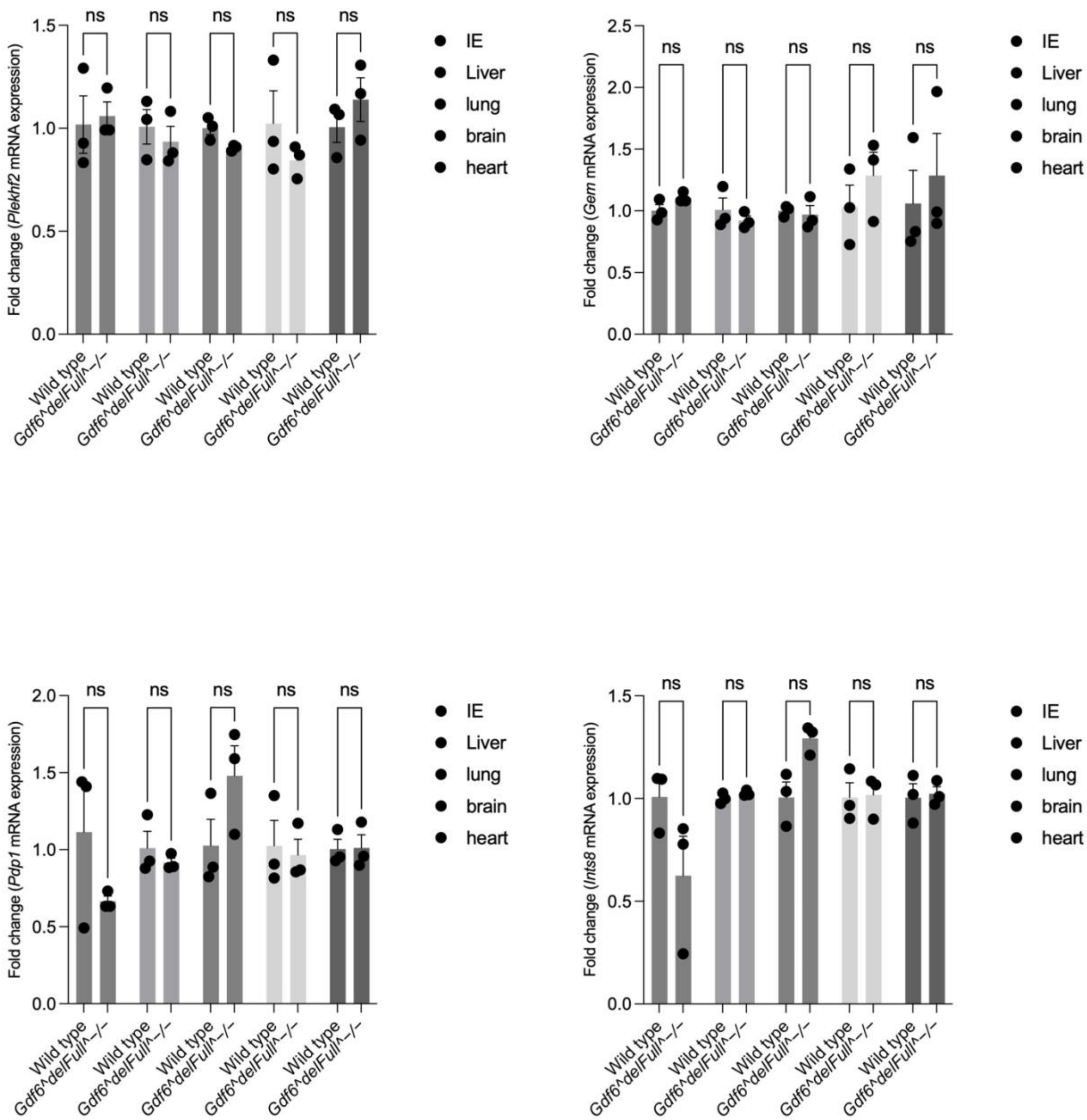

Figure S1: RT-PCR analysis of *Gdf6* and neighboring gene expression in P18 mouse tissues. qRT-PCR was performed on Inner ear, heart, and brain RNA from wild-type (WT) and *Gdf6^delFull^-/-* mice. Amplicons for *Gdf6, Plekhf2, GEM*, *Pdp1 and Ints8* are shown. Expression was normalized to *HPRT1*. *Gdf6* expression is present in *Gdf6^delFull^-/-* mice Inner-ear (IE), liver, lung, brain, and heart, indicating preservation of local gene regulation. The data represent three biological replicates. Results are expressed as Mean ± SEM. Statistical significance was determined using two-way ANOVA with Tukey's test for multiple comparisons ($p < 0.05$ considered significant).

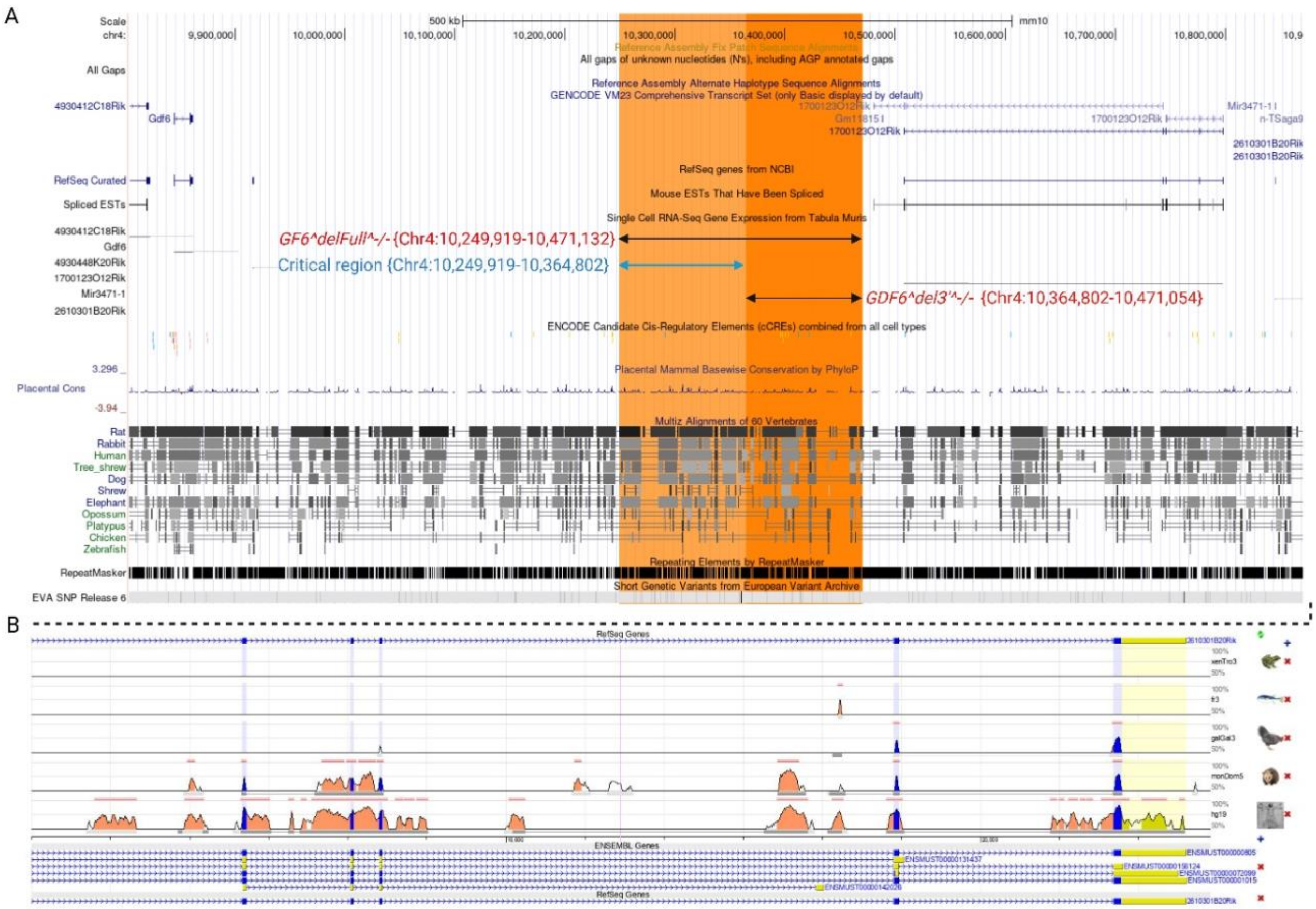

Figure S2: Genome browser views of *Gdf6* locus on mouse chromosome 4. (A) UCSC Genome Browser view (mm10 assembly) of the *Gdf6* locus on mouse chromosome 4, highlighting two overlapping deletions ("Gdf6^delFull^/^-" and "Gdf6^del3'^/^-") and the defined critical interval (orange shading). Tracks shown include annotated genes (RefSeq, GENCODE), spliced ESTs, and candidate cis-regulatory elements from ENCODE (cCREs). The placental-mammal PhyloP conservation track and Multiz 60-species alignment are displayed underneath, indicating evolutionary conservation across vertebrates. Repetitive elements (RepeatMasker) and short genetic variants (EVA SNP Release 6) are shown at the bottom. **(B)** ECR browser zoom on *1700123O12Rik*, illustrating individual peaks of alignment and further highlighting conserved segments across species.

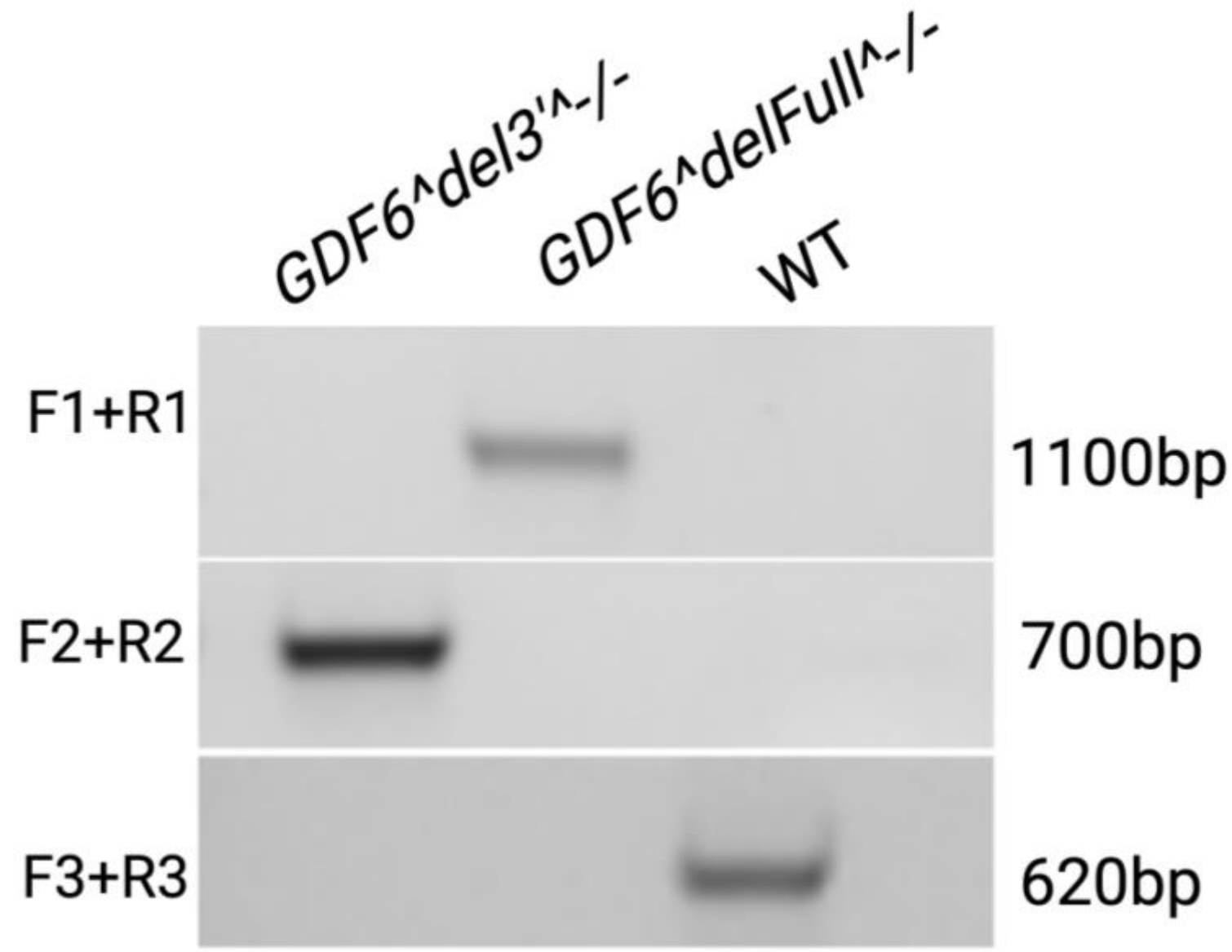

Figure S3: Characterization of genotypes in mouse. PCR-based genotyping of *GDF6* alleles in mice harboring either a partial 3′ deletion (*Gdf6^del3′*^−/−), a full locus deletion (*Gdf6^delFull*^−/−), or a wild-type (WT) allele. Each row shows PCR products amplified with a distinct primer pair (F1+R1, F2+R2, or F3+R3), and the expected product sizes (in bp) are indicated on the right. In the top panel, the ~1100 bp band (F1+R1) appears for the partial and full deletion genotypes, confirming the rearranged locus. In the middle panel, the ~700 bp band (F2+R2) is amplified specifically from the partial 3′ deletion allele. In the bottom panel, the ~620 bp band (F3+R3) is visible in the wild-type sample, indicating the intact *Gdf6* locus.

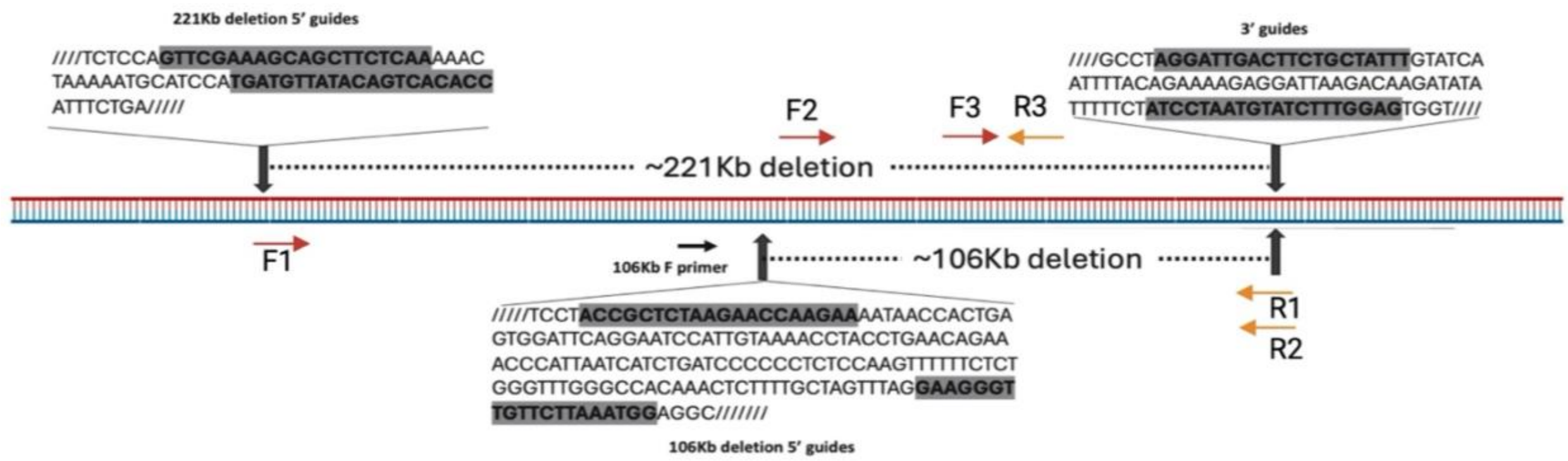

Figure S4: Schematic overview of the CRISPR/Cas9 strategy used to generate two large genomic deletions (~221 kb and ~106 kb). The top portion illustrates the ~221 kb deletion, showing the 5′ and 3′ guide RNA target sites (dark-shaded boxes) and the approximate genomic region (blue/red lines). Arrows labeled F1, F2, and F3 indicate forward primers used to screen for successful deletion, while R3 marks a reverse primer site. The dotted lines represent the intended deletion boundaries. The bottom portion shows the ~106 kb deletion design with its own 5′ guide RNA target sites (dark-shaded boxes) and an additional reverse primer pair (R1, R2) for PCR-based verification. Forward primer "106 kb F" is indicated by an arrow above the locus. Sequences in shaded boxes are representative portions of the gRNA target sites. Black arrowheads highlight the nuclease cut sites leading to the excision of the intervening genomic segments.

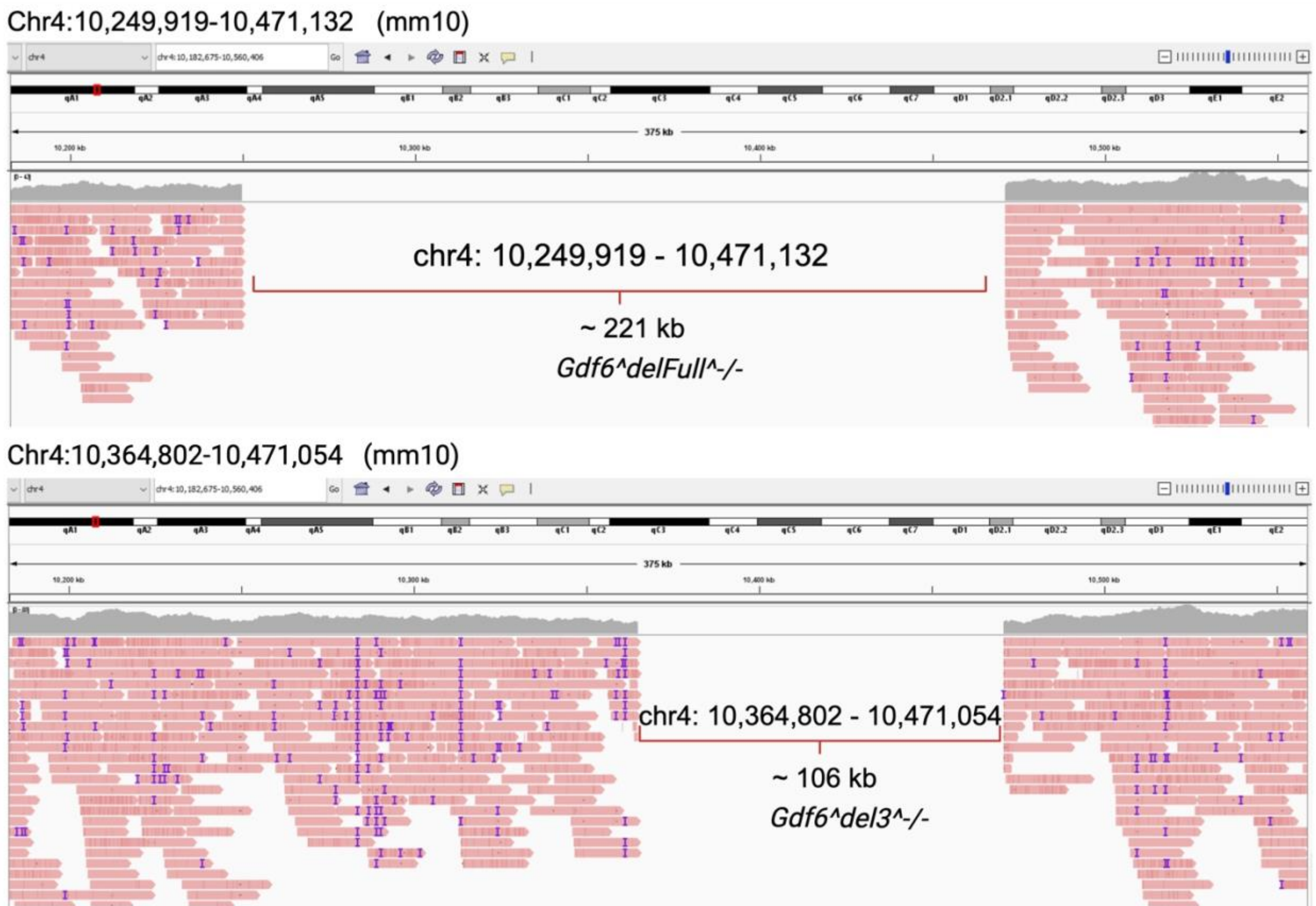

Figure S5: Identification of deletion breakpoints in a CRISPR/Cas9-generated knockout mouse model using long-read sequencing. (Top panels) Integrative Genomics Viewer (IGV) screenshots show long-read sequencing alignments (PacBio reads) from *Gdf6^delFull^-/- and Gdf6^del3'^-/-* mouse showing a 221,135 bp (chr 4:10,249,919–10,471,132 (mm10)}, and 106,252 bp deletion {chr4:10,364,802–10,471,054 (mm10)}, respectively. The critical region, defined as Chr4:10,249,919–10,364,802 (114,884 bp) in this study, is covered in *Gdf6^delFull^-/-,* but not in *Gdf6^del3'^-/-*.

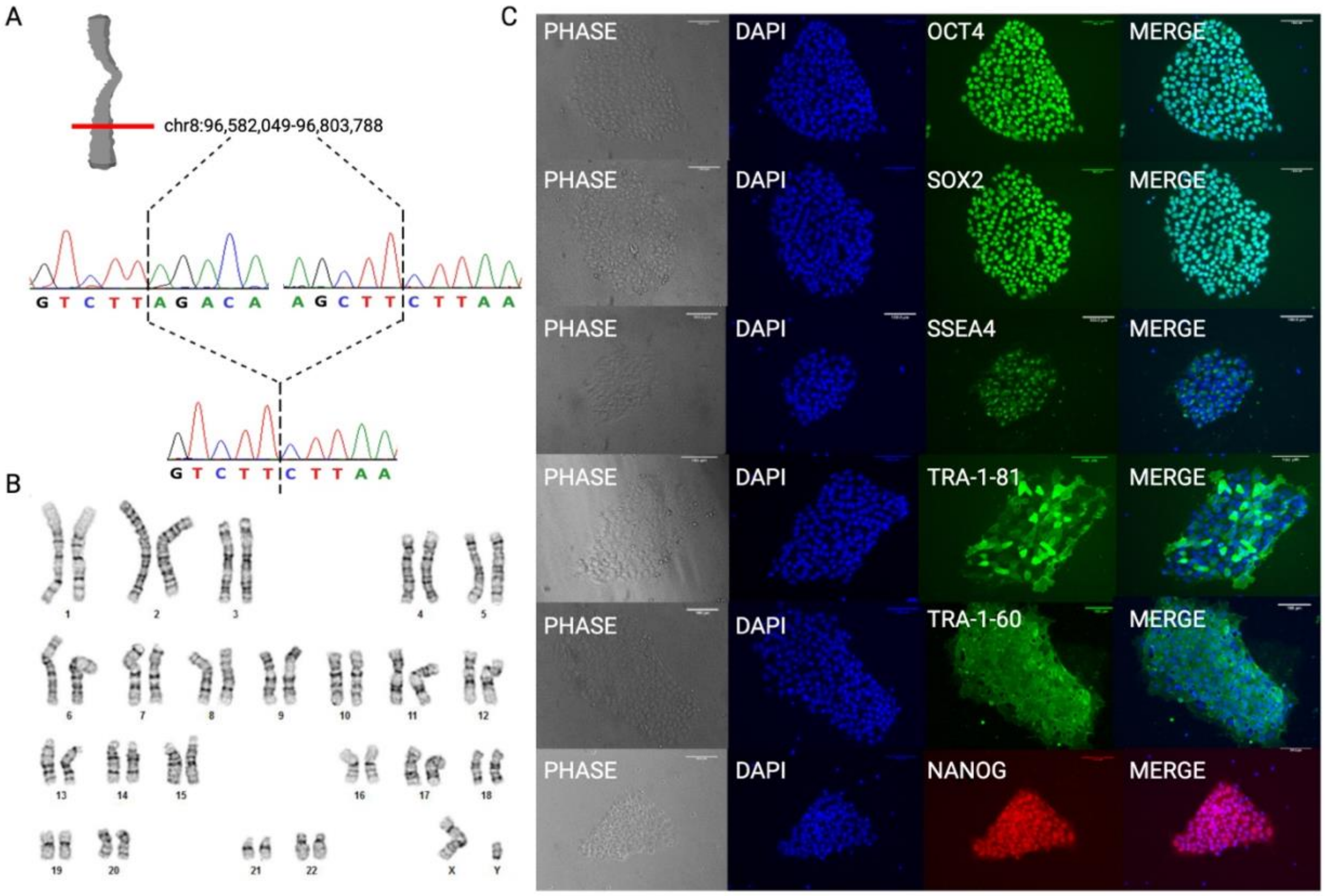

Figure S6: Characterization of patient-derived Induced Pluripotent Stem Cells (IPSCs). (A) Chromosomal localization and Sanger sequencing analysis of genomic deletion on chromosome 8 (chr8:96,582,049–96,803,788). **(B)** Karyotype analysis showing normal chromosomal integrity of the IPSC line. All 23 pairs of chromosomes (22 autosomes and one sex chromosome pair) are intact and properly aligned, confirming chromosomal stability. **(C)** Immunofluorescence staining of IPSC for pluripotency markers. Cells were stained with DAPI (blue) to visualize nuclei and probed for expression of pluripotency-associated proteins: OCT4, SOX2, SSEA4, TRA-1-81, TRA-1-60 (green), and NANOG (red). Merged images show co-localization of markers with nuclear staining, confirming pluripotent identity. Phase contrast images indicate typical colony morphology.

A

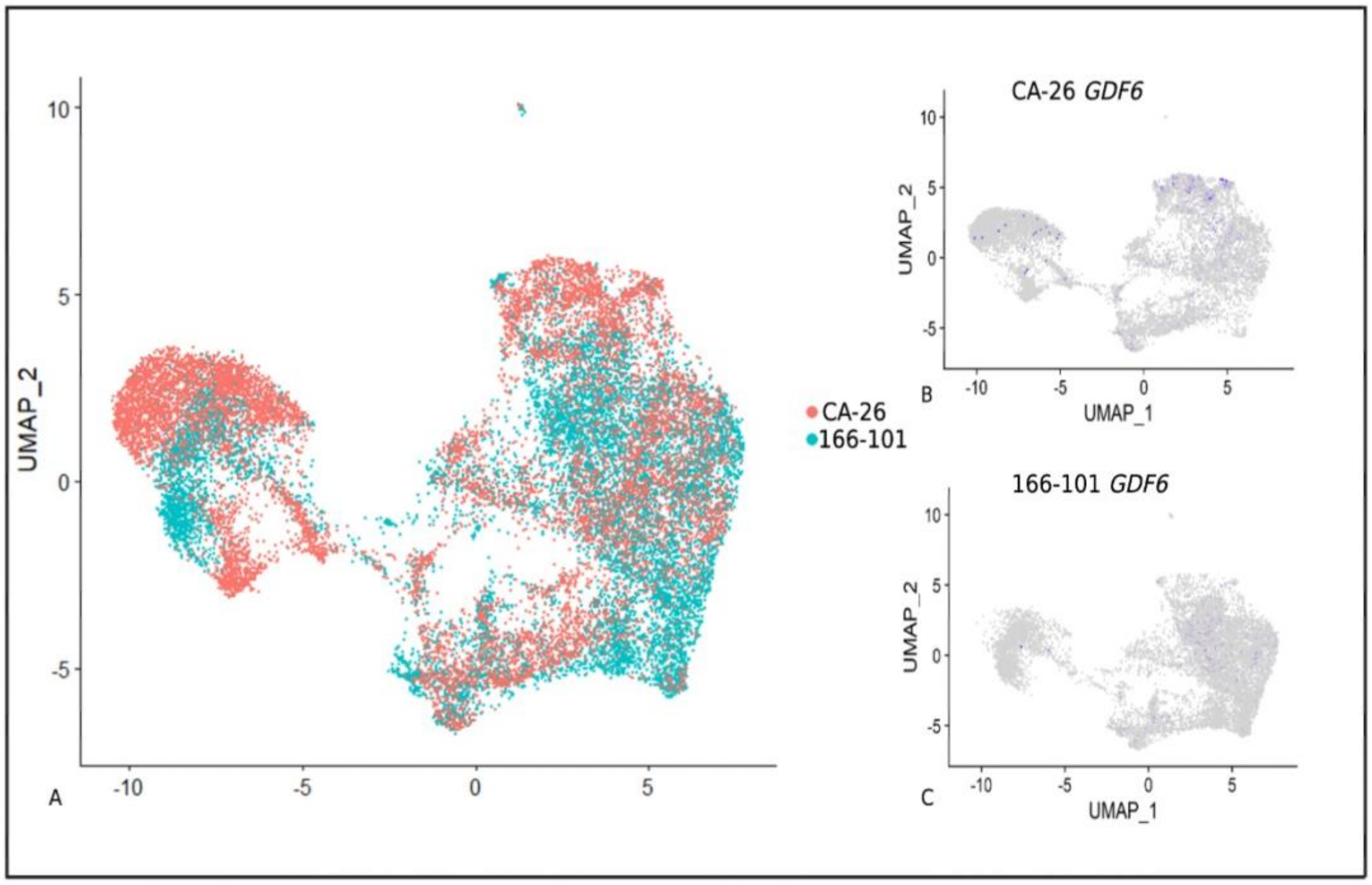

B

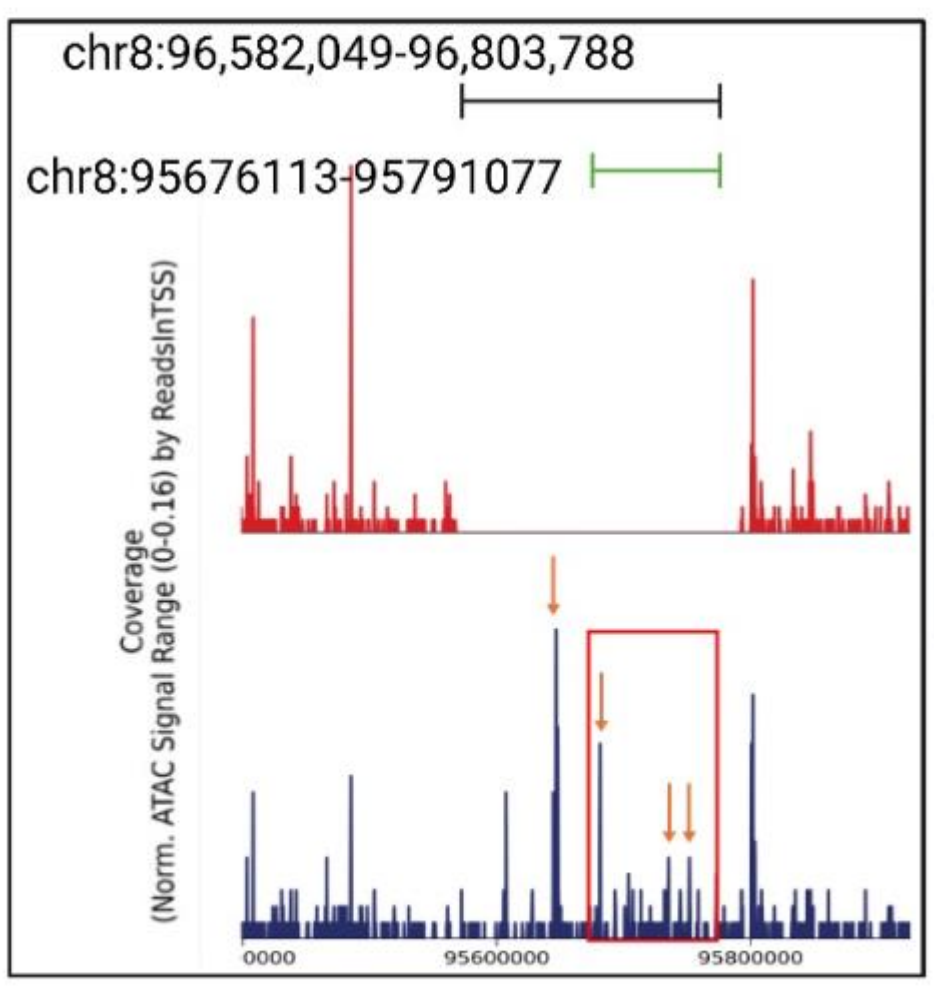

C

	start	end	log2FC	FC
**chr8**	95648167	95648667	-5.29192	-39.1765
**chr8**	95681386	95681886	-5.38172	-41.6927
**chr8**	95732244	95732744	-3.7005	-13.0006
**chr8**	95758585	95759085	-5.92763	-60.8688

Figure S7: Characterization of *GDF6* expression and chromatin accessibility in patient versus control cells by scRNA-seq and scATAC-seq. (A) UMAP visualization of single-cell RNA-seq data from two cell lines**:** control CA-26 (red) and patient 166-101 (teal). Each point represents a single cell, and clustering patterns reflect transcriptional similarities. (Right) Separate UMAP plots highlight *GDF6* expression in each cell line. Purple dots mark cells with detectable *GDF6* transcripts, illustrating the distribution of *GDF6*$^{+}$ cells across the two main UMAP clusters. **(B)** Normalized scATAC-seq coverage tracks spanning the *GDF6* locus on chromosome 8 (coordinates shown above). The top (red) track corresponds to patient (166-101), while the bottom (blue) track corresponds to control (CA-26). Black bars indicate the broader genomic region, and green bars indicate a smaller critical region for cochlear development. Orange arrows and the red box highlight putative regulatory peaks (regions of open chromatin) that may underlie the observed differences in *GDF6* expression between the two lines. **(C)** The significantly altered scATAC-seq peaks in the region downstream of *GDF6* between patient versus control samples are listed in the table. The peak containing a known CTCF-binding site is highlighted.

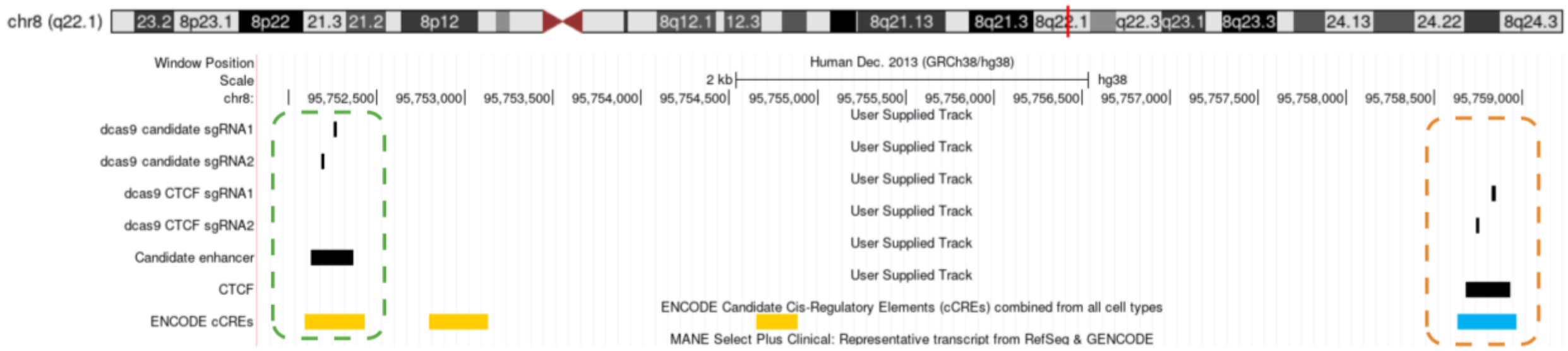

Figure S8: Genomic view of the targeted *GDF6* regulatory regions on chromosome 8q22.1. UCSC Genome Browser snapshot (hg38) showing the location of CD2-dCas9-CTCF sgRNAs and dCas9-Candidate, the candidate enhancer, predicted CTCF site, and overlapping ENCODE cCREs within the *GDF6* regulatory region. Dashed boxes highlight the two targeted subregions examined in this study, as shown in Figure 3.

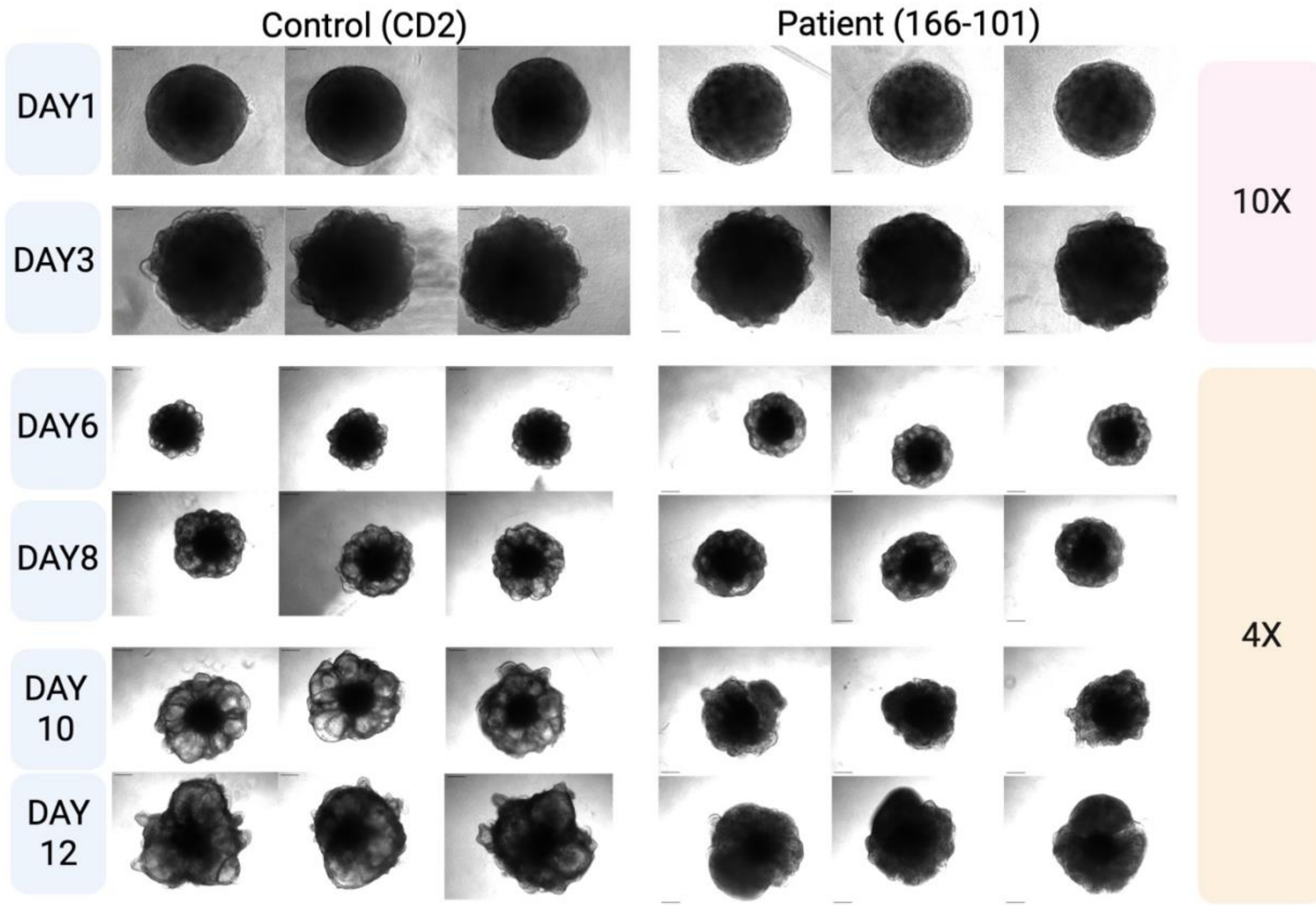

Figure S9: Morphological progression of inner ear organoids derived from control and patient iPSCs. Representative bright-field images of inner ear organoids derived from control (CD2) and patient (166-101) iPSCs from Day 1 to Day 12. Control organoids show progressive enlargement and increase peripheral budding, while patient organoids display comparatively reduced structural expansion over time. Images are at 10× (Day 1–3) and 4× (Day 6–12).

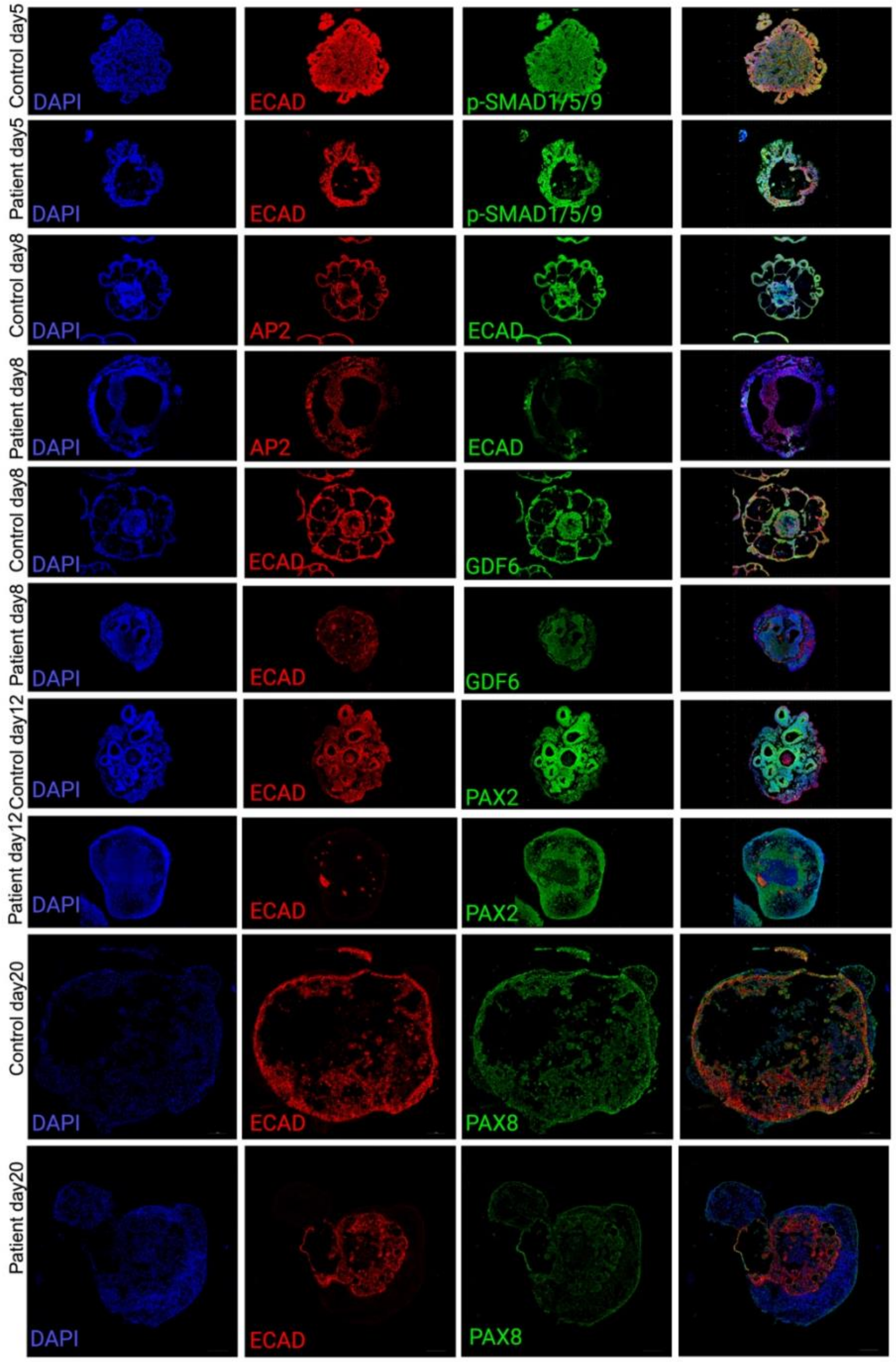

Figure S10: Early otic induction in control and patient 166-101 organoids. Immunofluorescence of organoids at days 5, 8, 12, and 20 stained for DAPI, ECAD, and stage-specific markers (p-SMAD1/5/9, AP2, GDF6, PAX2, PAX8). Images were acquired at 20x magnification.

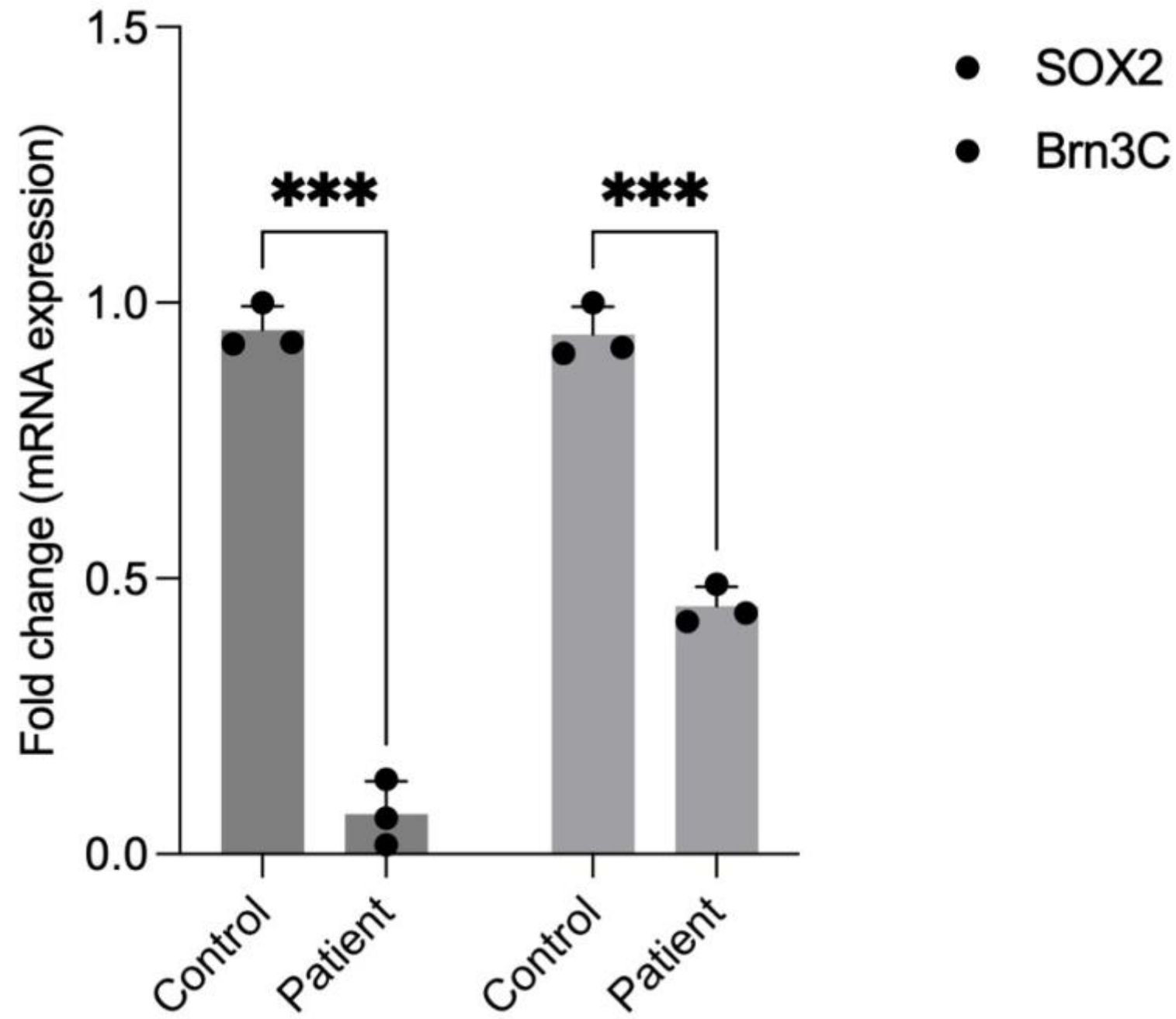

Figure S11: qRT-PCR analysis of sensory lineage markers in Day 55 inner ear organoids. *POU4F3* (*Brn3C*) and *SOX2* expressions were quantified in the Control (CD2) and in the Patient (166-101) organoids on Day 55. Values were normalized to *HPRT1* and are shown relative to the control. *POU4F3* (*Brn3C*) and *SOX2* levels were reduced in patient organoids. The data represent three biological replicates. Results are expressed as Mean ± SEM. Statistical significance was determined using one way ANOVA and using a Tukey post-hoc test ($p < 0.05$ considered significant).

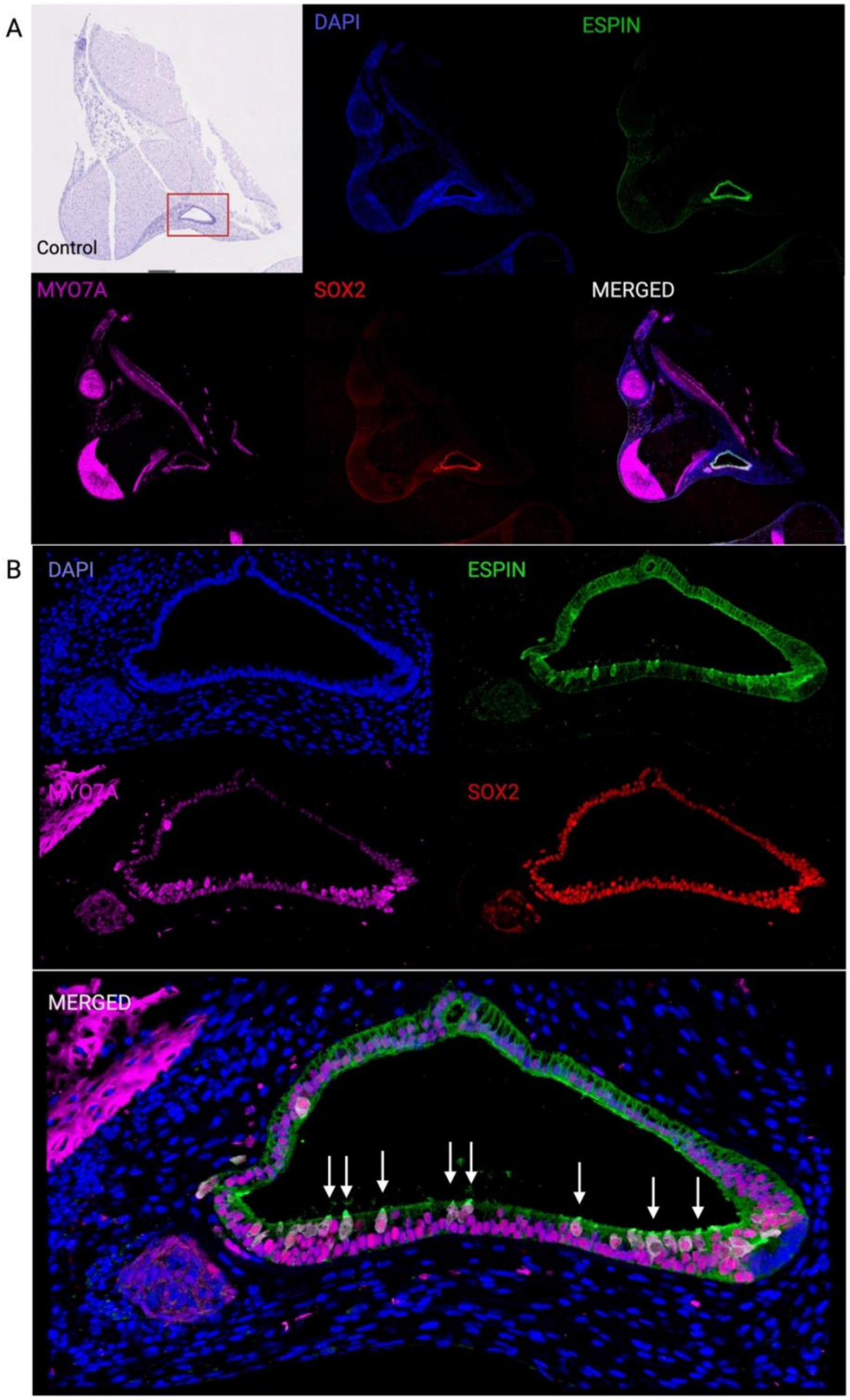

Figure S12: Sensory epithelium markers in control inner-ear organoids. (A) Day-60 organoid sections show an otic-vesicle-like epithelium stained for DAPI (blue), ESPN (green), MYO7A (magenta), and SOX2 (red). Merged panels outline organized sensory-epithelium-like domains. Images were acquired at 20x magnification**. (B)** Higher-magnification (40x) view of the boxed region. ESPN labels apical bundles, MYO7A marks hair-cell–like cells, and SOX2 identifies prosensory/supporting-cell-like populations. Arrows indicate MYO7A^{+}/SOX2^{+} cells consistent with early hair-cell differentiation.

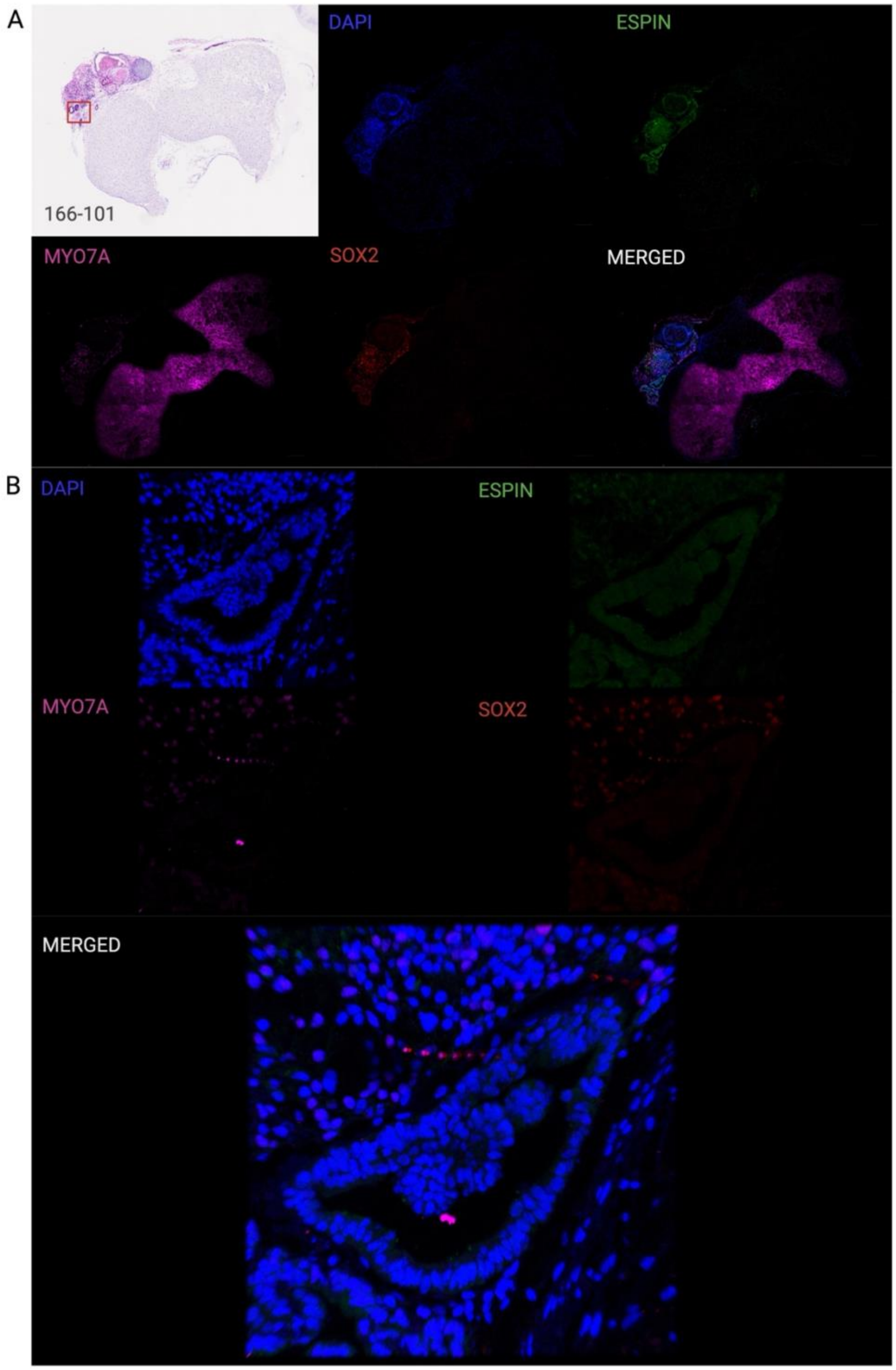

Figure S13: Sensory epithelium markers in inner-ear organoids derived from patient 166-101. (A) Day-60 inner-ear organoid section from patient 166-101 shows an otic-vesicle-like structure (H&E, left; boxed region) and the corresponding immunofluorescence for DAPI (blue), ESPN (green), MYO7A (magenta), and SOX2 (red). The merged image highlights a missing sensory-epithelium-like domain within the organoid. Images were acquired at 20x magnification**. (B)** Higher-magnification (40x) view of the boxed region. ESPN does not stain the apical epithelial surface; little to no SOX2 and MYO7A are visible in the merged panel.

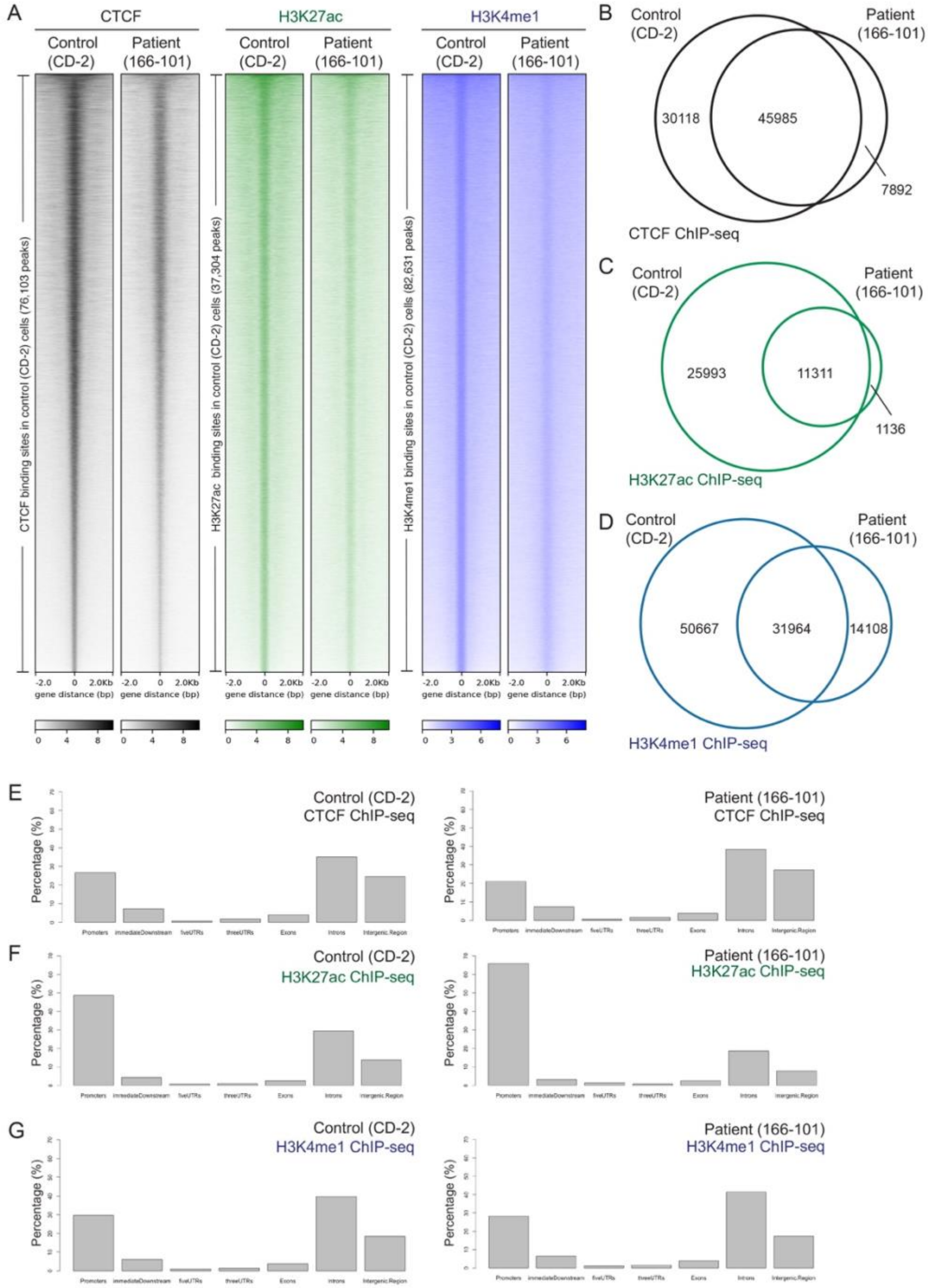

Figure S14: Chromatin landscape comparison between control and patient-derived CD2$^{(dcas9)}$ cells using ChIP-seq. (A) ChIP-seq profiling of CTCF, H3K27ac, and H3K4me1 was performed in control (CD-2) and patient (166-101) iPSC-derived cells to assess alterations in chromatin architecture. (A) Heatmaps display normalized ChIP-seq signal intensity centered on peak regions (±2 kb) for each factor, with peaks called from control cells. CTCF binding shows reduced signal in patient cells relative to control, while active enhancer marks H3K27ac and H3K4me1 also demonstrate altered enrichment patterns. **(B–D)** Venn diagrams illustrate the overlap and uniqueness of peaks between genotypes, revealing substantial differences in binding profiles: patient cells display a marked reduction in CTCF and H3K27ac peaks. In contrast, H3K4me1 peaks are partially preserved but still divergent. **(E–G)** Genomic distribution of peaks across annotated features shows a redistribution of binding in patient cells, particularly a shift in enhancer-associated marks away from promoter regions and toward intergenic and intronic regions. These results indicate broad chromatin remodeling in patient cells that may reflect disrupted transcriptional regulation and altered enhancer activity.

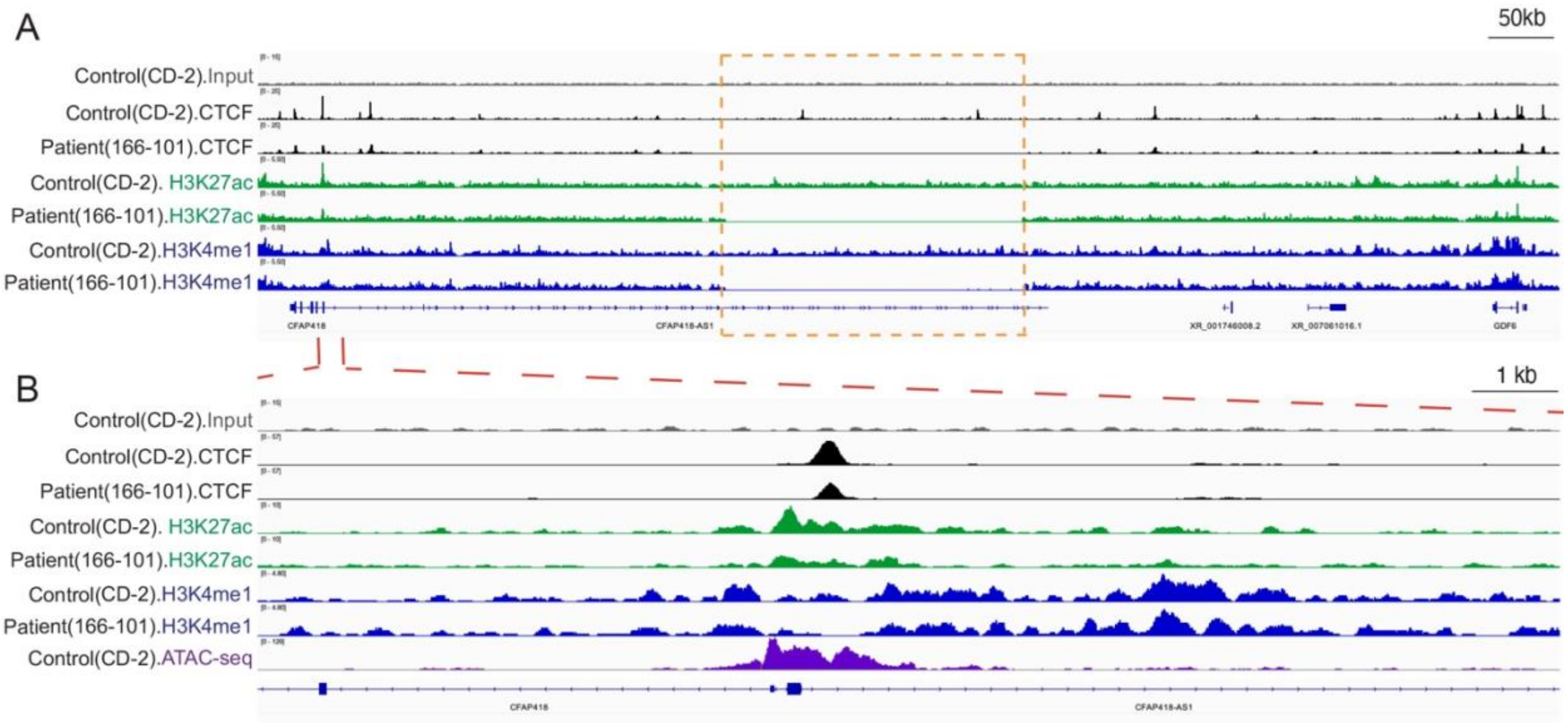

Figure S15: Disruption of local chromatin architecture in patient-derived IEO and CD2 IEO at the *CFAP418-AS1* locus. (A) Genome browser tracks show ChIP-seq signal across a ~300 kb region on chromosome 8 encompassing the *GDF6* gene and neighboring loci in control (CD-2) and patient (166-101) cells. Tracks represent binding profiles for CTCF (black), H3K27ac (green), and H3K4me1 (blue), as well as input controls. Compared to control, patient IEO exhibit a reduction in CTCF occupancy and altered enrichment of enhancer-associated marks H3K27ac and H3K4me1, suggesting local chromatin remodeling in the patient context.
(B) A zoomed-in view of the region highlighted in panel A, centered on the *CFAP418-AS1* locus, reveals a strong CTCF peak and enhancer signature (H3K27ac and H3K4me1) in control IEO that are diminished in patient IEO. ATAC-seq data (purple) from control IEO indicate this region is an accessible chromatin site, consistent with a putative regulatory element.

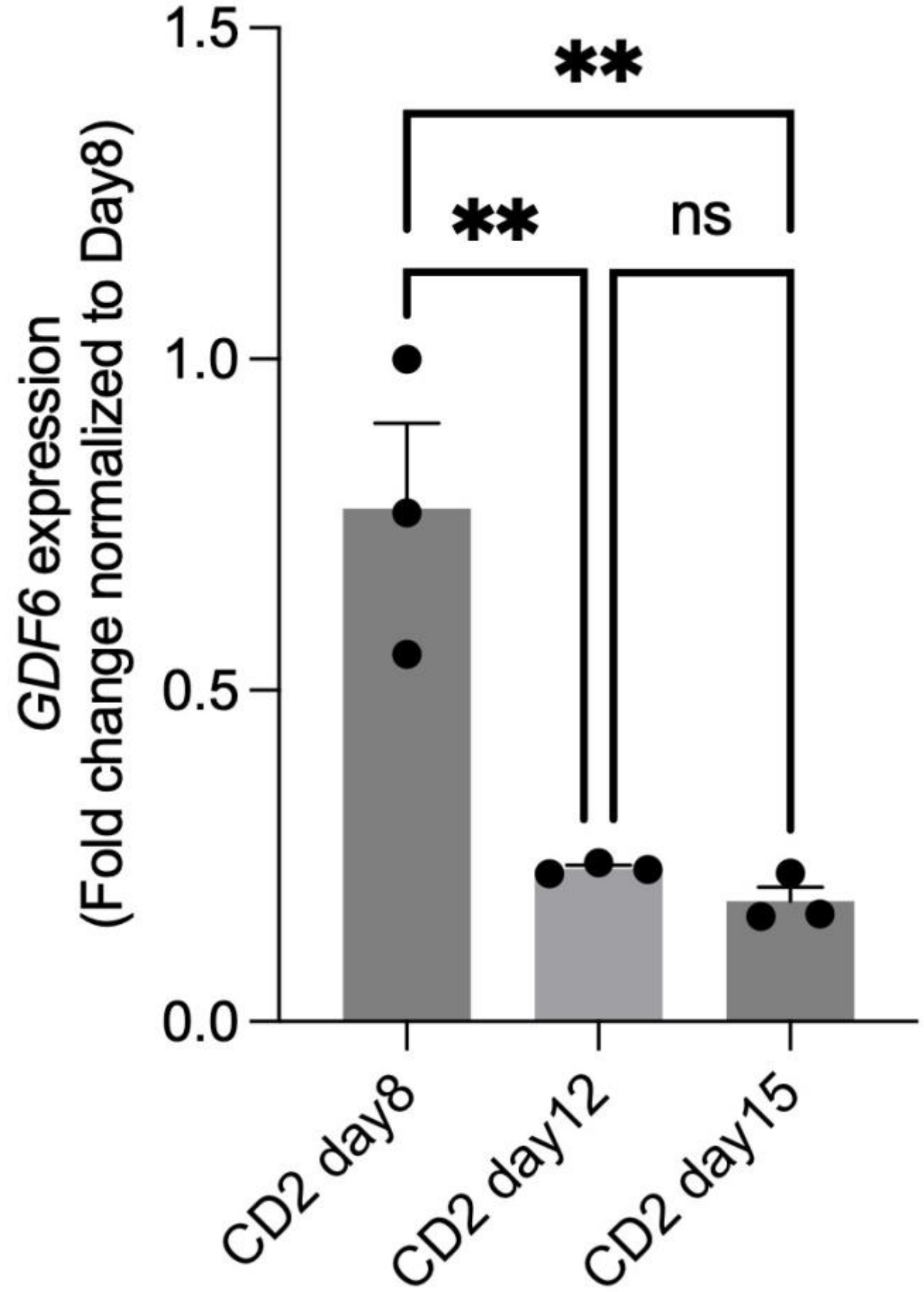

Figure S16: qRT-PCR analysis of *GDF6* expression in control iPSC-derived otic organoids relative to Day 8. qRT-PCR quantification of *GDF6* mRNA levels in control (CD2) organoids during early otic induction. Expression is normalized to *HPRT*1 and shown relative to Day 8. The data represent three biological replicates. Results are expressed as Mean ± SEM. Statistical significance was determined using one-way ANOVA and using a Tukey post-hoc test ($p < 0.05$ considered significant).

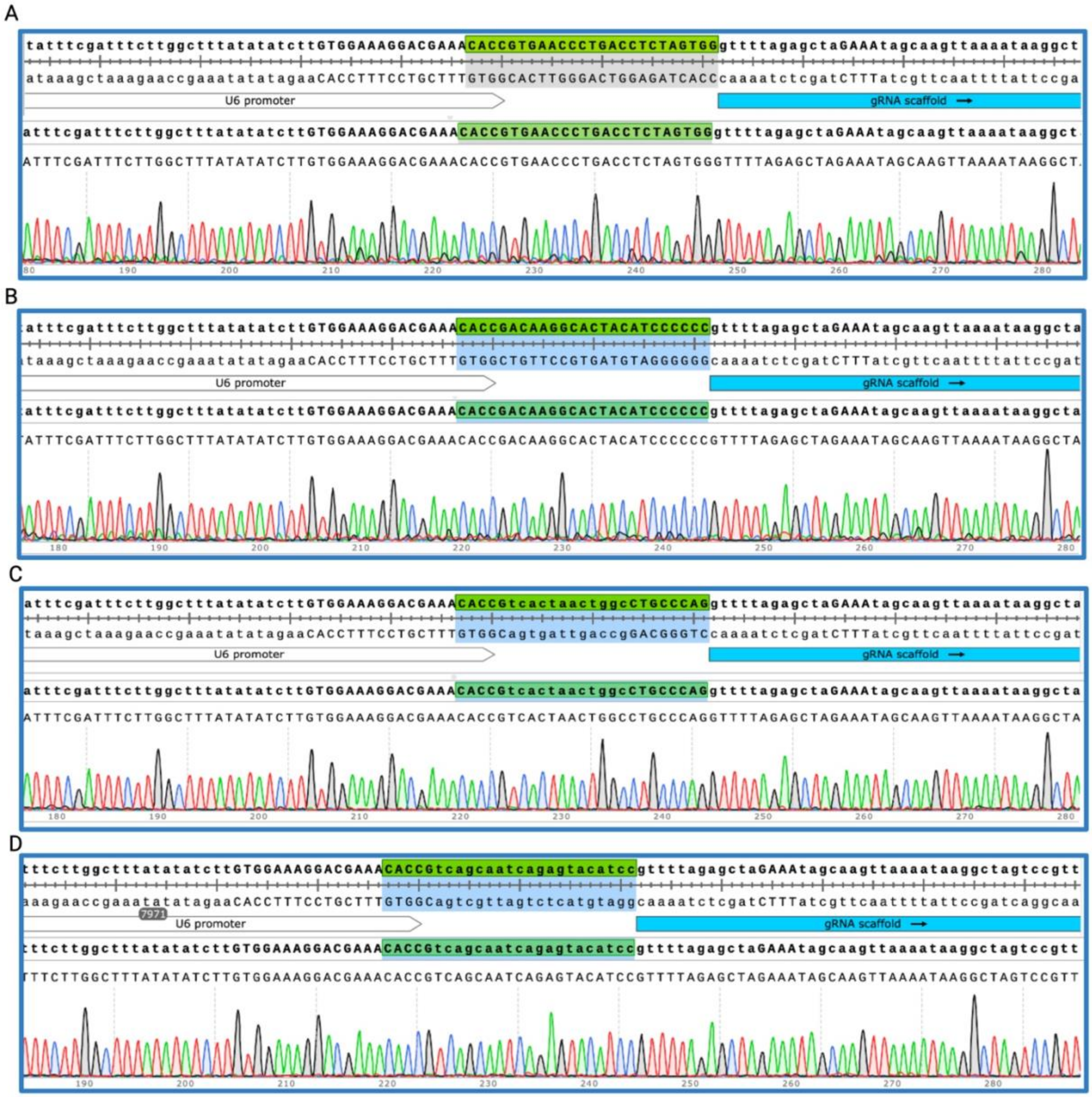

Figure S17: Sanger traces for LentiGuide vectors showing successful cloning of sgRNA sequences. Sanger sequencing chromatograms confirm the proper insertion of guide RNA sequences into the LentiGuide vector backbone. Panels **(A–D)** show the aligned nucleotide sequence of the U6 promoter, sgRNA target sequence (highlighted in green), and the gRNA scaffold (blue). Each trace demonstrates clean base calling with correct junctions, indicating successful cloning of distinct guide sequences into the vector. The U6 promoter drives the expression of the inserted guide RNAs for downstream CRISPRi perturbations.

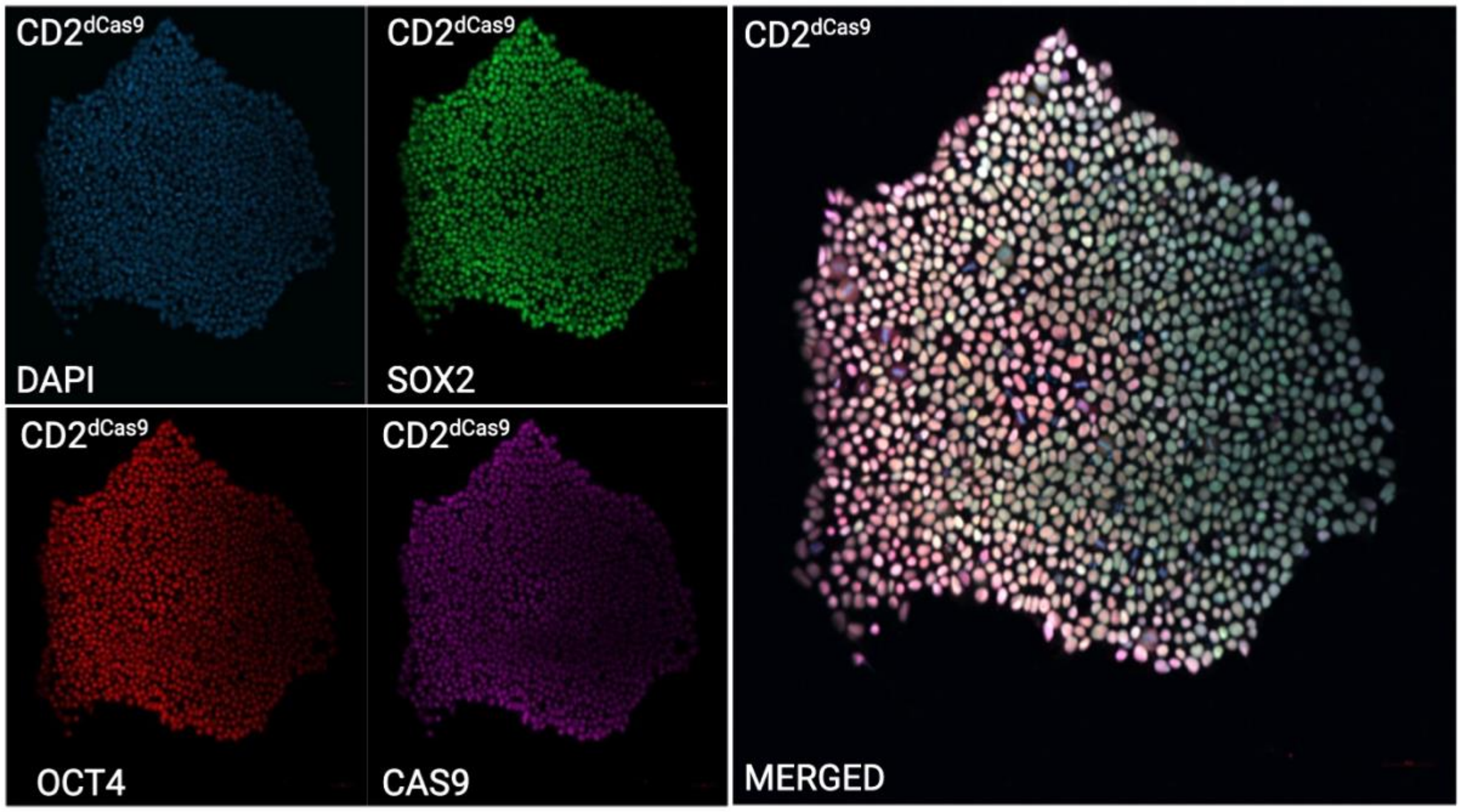

Figure S18: Immunofluorescence staining of CD2^{dDcas9} human pluripotent stem cells expressing dCas9. Representative confocal images (20x magnification) show the expression of pluripotency markers SOX2 (green) and OCT4 (red), along with DAPI nuclear staining (blue). Cells also express dCas9 (magenta), indicating successful delivery and nuclear localization of the dCas9 protein. The merged panel (right) confirms the co-expression of all markers across a uniform colony, supporting the maintenance of pluripotency and the functionality of the dCas9 system in the CD2$^{(dDcas9)}$ cell line.

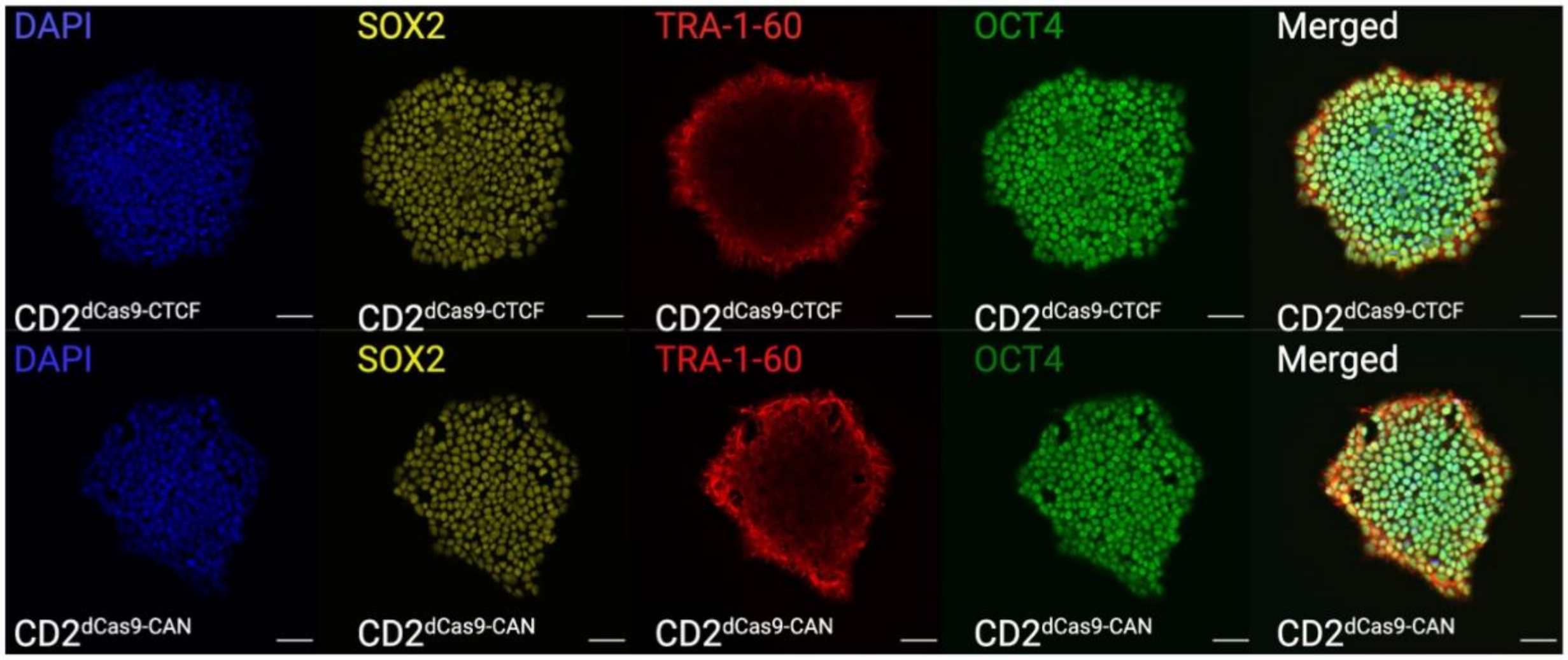

Figure S19: Pluripotency marker expression in CD2$^{dCas9\text{-}CTCF}$ and $^{CD2dCas9\text{-}CAN}$ iPSC lines. Immunofluorescence staining for DAPI, SOX2, TRA-1-60, and OCT4 reveals strong, comparable expression of pluripotency markers in both engineered iPSC lines. Merged images confirm the maintenance of colony morphology and the consistent distribution of markers. Images were acquired at 20x magnification.

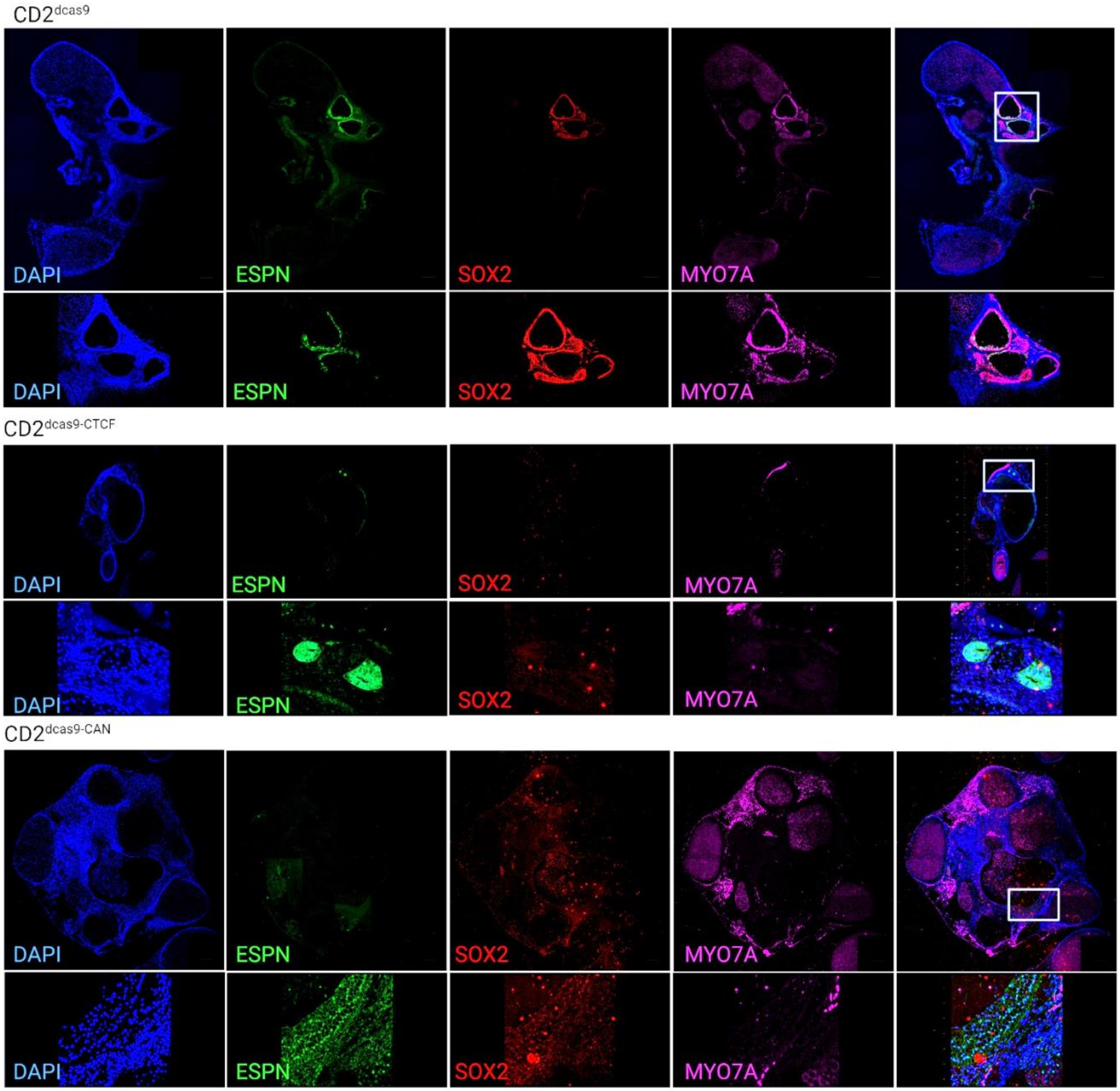

Figure S20: Characterization of inner-ear organoids by confocal imaging. Single-channel confocal images of figure 4 inner-ear organoids derived under CD2^{dCas9}, CD2$^{dCas9-CTCF}$, and CD2$^{dCas9-CAN}$ conditions. Organoids were stained for DAPI, ESPN, SOX2, and MYO7A.

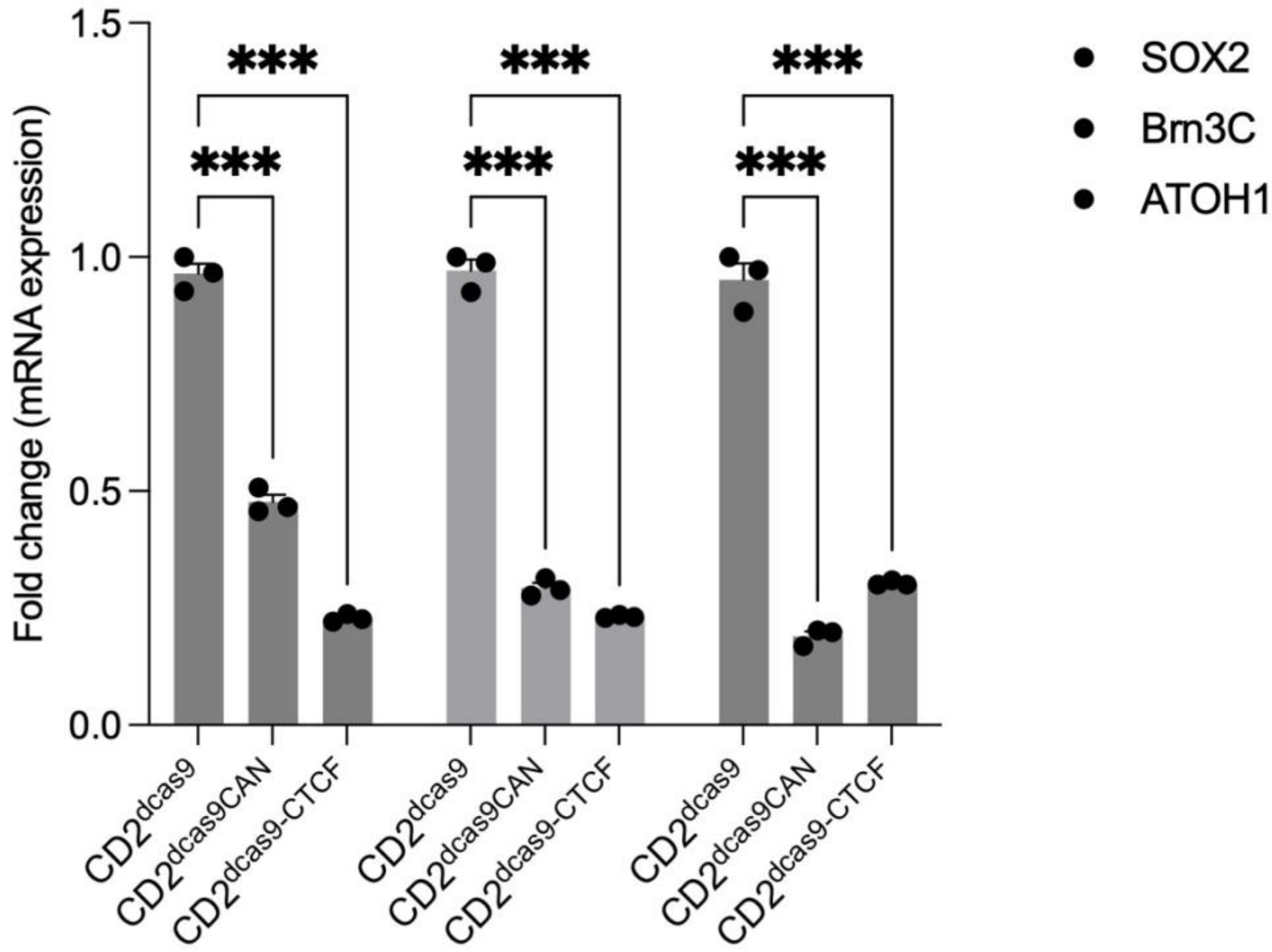

Figure S21: qRT-PCR analysis of sensory lineage markers in Day 55 inner ear organoids. *SOX2 and POU4F3 (Brn3C)* expressions were quantified in $CD2^{dcas9}$, $CD2^{dcas9\text{-}CTCF}$ and $CD2^{dcas9\text{-}CAN}$ organoids on Day 55. Values were normalized to *HPRT1* and are shown relative to the control. The data represent three biological replicates. Results are expressed as Mean ± SEM. Statistical significance was determined using one-way ANOVA and using a Tukey post-hoc test ($p < 0.05$ considered significant).

Supplemental Table 1: Sequences of sgRNA used to generate cis-regulatory deletion mice *GDF6^del3'^-/-* and *GDF6^delFull^-/-.*

Name	sgRNA Guide sequences
221kb 5' guide 1	TTGAGAAGCTGCTTTCGAAC
221kb 5' guide 2	GGTGTGACTGTATAACATCA
106kb 5' guide 1	TTCTTGGTTCTTAGAGCGGT
106kb 5' guide 2	GAAGGGTTGTTCTTAAATGG
3' guide 1	ATCCTAATGTATCTTTGGAG
3' guide 2	AAATAGCAGAAGTCAATCCT

Supplemental Table 2: Primer information for cloning sgRNA in Lentiviral backbone.

Name	Target site	Sequence
CTCF-Assembly1-R	chr8:95758681-95758931	AAACCCACTAGAGGTCAGGGTTCAC
CTCF- Assembly1-F	chr8:95758681-95758931	CACCGTGAACCCTGACCTCTAGTGG
CTCF-Assembly2-R	chr8:95758681-95758931	AAACGGGGGGATGTAGTGCCTTGTC
CTCF- Assembly2-F	chr8:95758681-95758931	CACCGACAAGGCACTACATCCCCCC
CAN-Assembly1-R	chr8:95752126-95752366	AAACCTGGGCAGGCCAGTTAGTGAC
CAN- Assembly1-F	chr8:95752126-95752366	CACCGTCACTAACTGGCCTGCCCAG
CAN-Assembly2-R	95752126-95752366	AAACGGATGTACTCTGATTGCTGAC
CAN- Assembly2-F	95752126-95752366	CACCGTCAGCAATCAGAGTACATCC

Supplemental Table 3: Primer information for qRT-PCR.

Name	Target	RefSeq	IDT assay ID	Sequences (5'-3')
Gdf6	mouse	NM_013526	Mm.PT.58.9175578	AGTCTTCCAAGTCAGCT AATACG<br>TCTTCTTTGTCTGAGAGTGTGG
Gem	mouse	NM_010276	Mm.PT.58.16213709	GTCGAGAAGTGTCTGTGTCAG<br>ATGCCCTCAAACAGTTCCTT
Pdp1	mouse	NM_001098231	Mm.PT.58.7065334	GTGATGACAGGAGAATGTGTGT<br>CATCAGAGAACAGTGGCAGAC
Plekhf2	mouse	NM_ 175175	Mm.PT.58.11463811	CGACTAGGAGGACTCGCA<br>CGTCTAGTATTCGCCTCACTG
Hprt	mouse	NM_013556	Mm.PT.39a.22214828	CCCCAAAATGGTTAAGGTTGC<br>AACAAAGTCTGGCCTGT ATCC
Ints8	mouse	NM_178112	Mm.PT.58.5615433	GATATCACAGCCGAGCACAT<br>CTGAGTGTATATCGAACTAGCTCT
HPRT	human	NM_000194	Hs.PT.58v.45621572	TTGTTGTAGGATATGCCCTTGA<br>GCGATGTCAATAGGACTCCAG
PAX2	human	NM_003990	Hs.PT.58.27416693	AAACGACAGAACCCGACTATG<br>GGTGGAAAGGCTGCTGAA
ECAD	human	NM_004360	Hs.PT.58.45747629	CTGCTCTTGCTGTTTCTTCG<br>CTGTGCAGCTGGCTCAA
POU4F3	human	NM_002700	Hs.PT.58.39481748.g	GCTGCAAGAACCCAAA TTCTC<br>GCTCTCATCAAAGCTTCCAAA
ATOHl	human	NM_005172	Hs.PT.56a.26560683.g	CAATGTTATCCCGTCGTTCAAC<br>GGACAAGGCGTTGATGTAGA
SOX2	human	NA	NA	AGACGCTCATGAAGAAGGATAAG<br>CCGCTCGCCATGCTATT
